# GABAergic modulation of olfactomotor transmission in lampreys

**DOI:** 10.1371/journal.pbio.2005512

**Published:** 2018-10-04

**Authors:** Gheylen Daghfous, François Auclair, Felix Clotten, Jean-Luc Létourneau, Elias Atallah, Jean-Patrick Millette, Dominique Derjean, Richard Robitaille, Barbara S. Zielinski, Réjean Dubuc

**Affiliations:** 1 Groupe de Recherche sur le Système Nerveux Central, Département de neurosciences, Université de Montréal, Montréal, Québec, Canada; 2 Groupe de Recherche en Activité Physique Adaptée, Département des sciences de l'activité physique, Université du Québec à Montréal, Montréal, Québec, Canada; 3 Department of Biological Sciences, University of Windsor, Windsor, Ontario, Canada; 4 Great Lakes Institute for Environmental Research, University of Windsor, Windsor, Ontario, Canada; University of Lausanne, Switzerland

## Abstract

Odor-guided behaviors, including homing, predator avoidance, or food and mate searching, are ubiquitous in animals. It is only recently that the neural substrate underlying olfactomotor behaviors in vertebrates was uncovered in lampreys. It consists of a neural pathway extending from the medial part of the olfactory bulb (medOB) to locomotor control centers in the brainstem via a single relay in the caudal diencephalon. This hardwired olfactomotor pathway is present throughout life and may be responsible for the olfactory-induced motor behaviors seen at all life stages. We investigated modulatory mechanisms acting on this pathway by conducting anatomical (tract tracing and immunohistochemistry) and physiological (intracellular recordings and calcium imaging) experiments on lamprey brain preparations. We show that the GABAergic circuitry of the olfactory bulb (OB) acts as a gatekeeper of this hardwired sensorimotor pathway. We also demonstrate the presence of a novel olfactomotor pathway that originates in the non-medOB and consists of a projection to the lateral pallium (LPal) that, in turn, projects to the caudal diencephalon and to the mesencephalic locomotor region (MLR). Our results indicate that olfactory inputs can induce behavioral responses by activating brain locomotor centers via two distinct pathways that are strongly modulated by GABA in the OB. The existence of segregated olfactory subsystems in lampreys suggests that the organization of the olfactory system in functional clusters may be a common ancestral trait of vertebrates.

## Author summary

Olfactory-induced behaviors (homing, food or mate searching, etc.) are crucial for the survival and reproduction of most animals. A neural substrate underlying odor-induced behaviors in vertebrates was recently uncovered using a basal vertebrate model: the lamprey. It consists of a neural pathway extending from the medial olfactory bulb, a first-order relay of olfactory information in the brain, to locomotor regions. Here, we investigated modulatory mechanisms acting on this neural pathway. We show that an inhibitory circuitry that releases the neurotransmitter GABA in the olfactory bulb strongly modulates motor responses to olfactory stimulation. We also discovered and characterized a novel olfactomotor pathway that originates in the non-medial olfactory bulb and consists of a projection to the lamprey olfactory cortex that, in turn, projects to locomotor regions. This discovery of a novel pathway linking olfactory and motor centers in the brain indicates that olfactory inputs can activate locomotor centers via two distinct pathways. Both pathways are strongly modulated by the neurotransmitter GABA in the olfactory bulb. The existence of segregated olfactory subsystems in lampreys sheds light on the evolution of olfactory systems in vertebrates.

## Introduction

Olfactory cues can trigger goal-directed locomotor behaviors, such as homing, predator avoidance, or food and mate searching [1–11]. It is only recently that the neural pathways and mechanisms involved in transforming olfactory inputs into locomotor behavior were characterized for the first time in a vertebrate species, the lamprey [12,13]. It consists of a specific neural pathway extending from a single glomerulus located in the medial part of the olfactory bulb (medOB) to the mesencephalic locomotor region (MLR), with a relay in the posterior tuberculum (PT) [12]. In all vertebrates, the MLR acts as a motor command center that controls locomotion via descending projections to brainstem reticulospinal (RS) neurons [14–22]. This olfactomotor pathway is present throughout the life cycle of lampreys, whether in larvae, newly transformed, parasitic, or spawning animals [12]. Yet, olfactory-induced motor behaviors can be life stage specific in lampreys. For instance, at the parasitic stage, lampreys feed on fish that they detect using olfactory cues [23]. Then, when sexually mature, the adults are attracted upstream by migratory pheromones released by larvae [24–26]. Once upstream, the females are attracted to males by sex pheromones [27,28].

The general organization of the lamprey olfactory system, from the periphery to the central nervous system (CNS), is very similar to that of other vertebrates. The peripheral olfactory organ is composed of a main olfactory epithelium and an accessory olfactory organ [29–31]. Axons from olfactory sensory neurons (OSNs) of the olfactory epithelium terminate in the olfactory bulb (OB). As in other vertebrates, the OB can be divided in two subregions, based on their inputs. The main olfactory bulb (MOB), which occupies the whole OB except its medial part (i.e., the medOB), receives inputs from the main olfactory epithelium. The medOB, on the other hand, receives inputs from OSNs located in the accessory olfactory organ [32–34]. The OB of vertebrates constitutes the primary olfactory center of the CNS and, as such, filters and actively shapes sensory inputs to secondary olfactory structures [35,36]. This processing of sensory inputs in the OB is driven by modulatory inputs coming from the numerous neurotransmitter systems present in the OB of vertebrates [37,38]. GABA is the main inhibitory neurotransmitter in the CNS, and numerous GABAergic processes are present in the OB of several vertebrate species [39–43]. GABAergic neurons of the OB are believed to play a critical role in olfactory processing by providing inhibition to the bulbar microcircuitry [44]. However, their effect on the outputs of the OB and ultimately on behavior is far less understood.

Here, we hypothesized that GABAergic neurons of the OB could play a significant role in modulating transmission in the olfactomotor pathway of lampreys. To address this, we used anatomical (tract tracing and immunohistochemistry) and physiological (intracellular recordings) techniques.

## Results

### GABAergic gating of olfactory inputs to RS neurons

**Fig 1:**
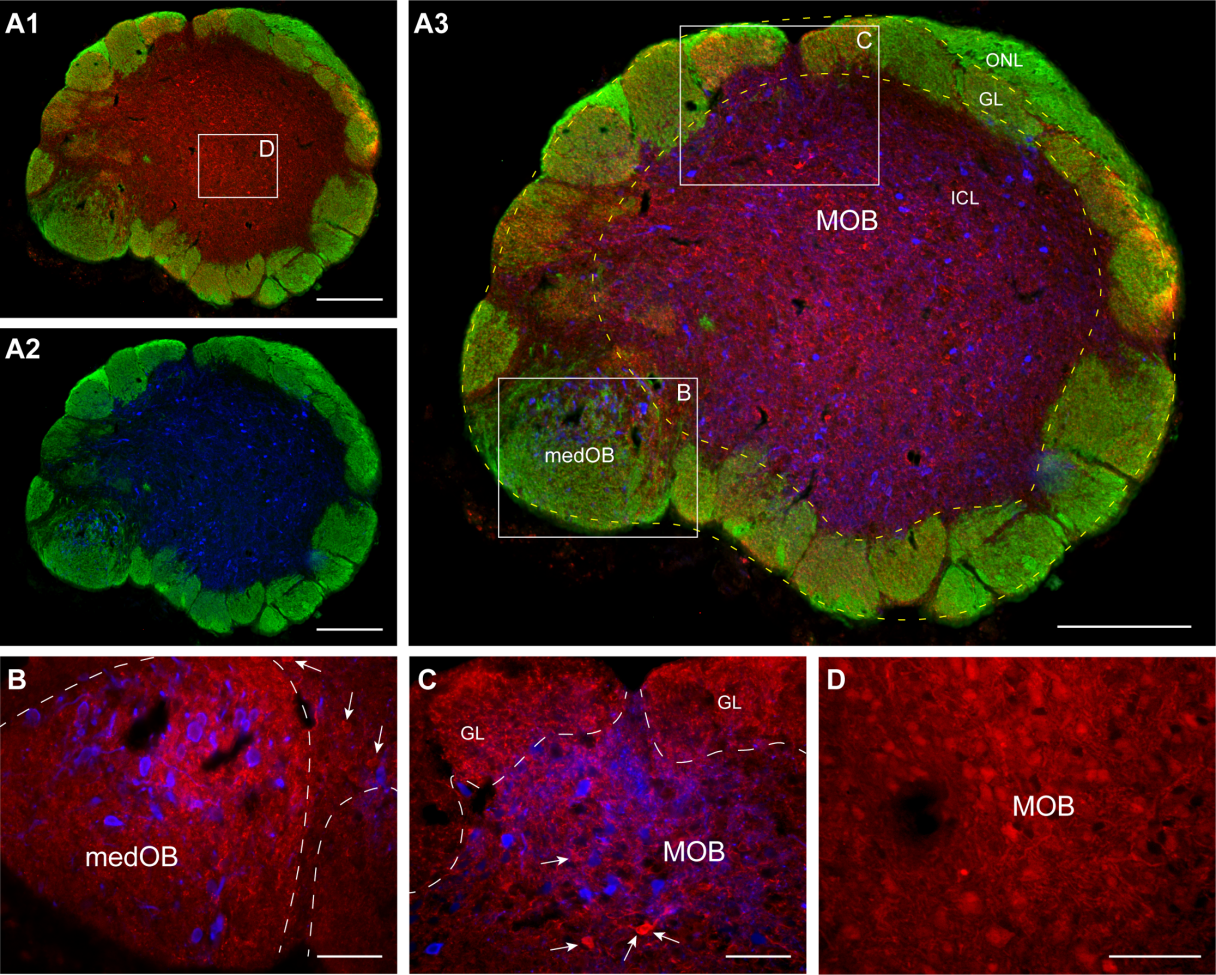
GABA-immunoreactive cell bodies and processes, as well as projection neurons in the olfactory bulb. (A) Low-power photomicrographs from a 25-μm-thick cross section of the olfactory bulb showing multiple labelings: binding of GSIB4 to olfactory primary afferents (green), GABA immunofluorescence (red), and retrograde labeling from injections in the PT and LPal (blue). (A1) The distribution of GABAergic neurons in relation to the GL. (A2) The distribution of projection neurons in relation to the GL. (A3) Merge of A1 and A2 showing some overlap in the distribution of the GABAergic and projection neurons populations. The white frames correspond to the approximate location of photomicrographs B to D. (B) High-power photomicrograph from a section just adjacent to the one illustrated in A, showing GABA processes in the medOB glomerulus. The projection neurons (blue) were labeled from an injection in the PT and are seen inside the medOB glomerulus. Arrows point at some GABA cell bodies lying outside of the glomerulus. The green fluorescence of olfactory primary afferents was omitted for clarity, but the contours of the glomerulus are indicated with a dashed line. (C) High-power photomicrograph from the section in A, showing GABA processes in the MOB GL and in the area where projection neurons (blue) are found. Those projection neurons were labeled from an injection in the LPal. Arrows point at some GABA cell bodies. The green fluorescence of olfactory primary afferents was omitted for clarity but the contours of the glomeruli are indicated with a dashed line. (D) Photomicrograph of the central region of the olfactory bulb showing numerous GABAergic neurons and processes. This thicker section was obtained from a different experiment, in which the immunofluorescence procedure was carried out on free-floating sections. Scale bars in A = 200 μm; scale bars in B-D = 50 μm. GL, glomerular layer; GSIB4, *Griffonia simplicifolia* isolectin B4; ICL, internal cell layer; LPal, lateral pallium; medOB, medial part of the olfactory bulb; MOB, main olfactory bulb, ONL, olfactory nerve layer; PT, posterior tuberculum.

The present study showed abundant GABAergic cell bodies and processes in the OB (*n* = 10 adult animals, Fig 1), thus confirming the findings of Meléndez-Ferro and colleagues [43]. GABAergic neurons were mainly observed in the central region of the OB (internal cell layer [ICL], Fig 1A and 1D and S1 Fig), where the most common OB interneuron type, the granule cell, was described [45]. GABAergic processes were found all over the OB, including in and around the glomeruli of both the MOB (Fig 1A and 1C) and the medOB (Fig 1A and 1B and S2 Fig).

**Fig 2:**
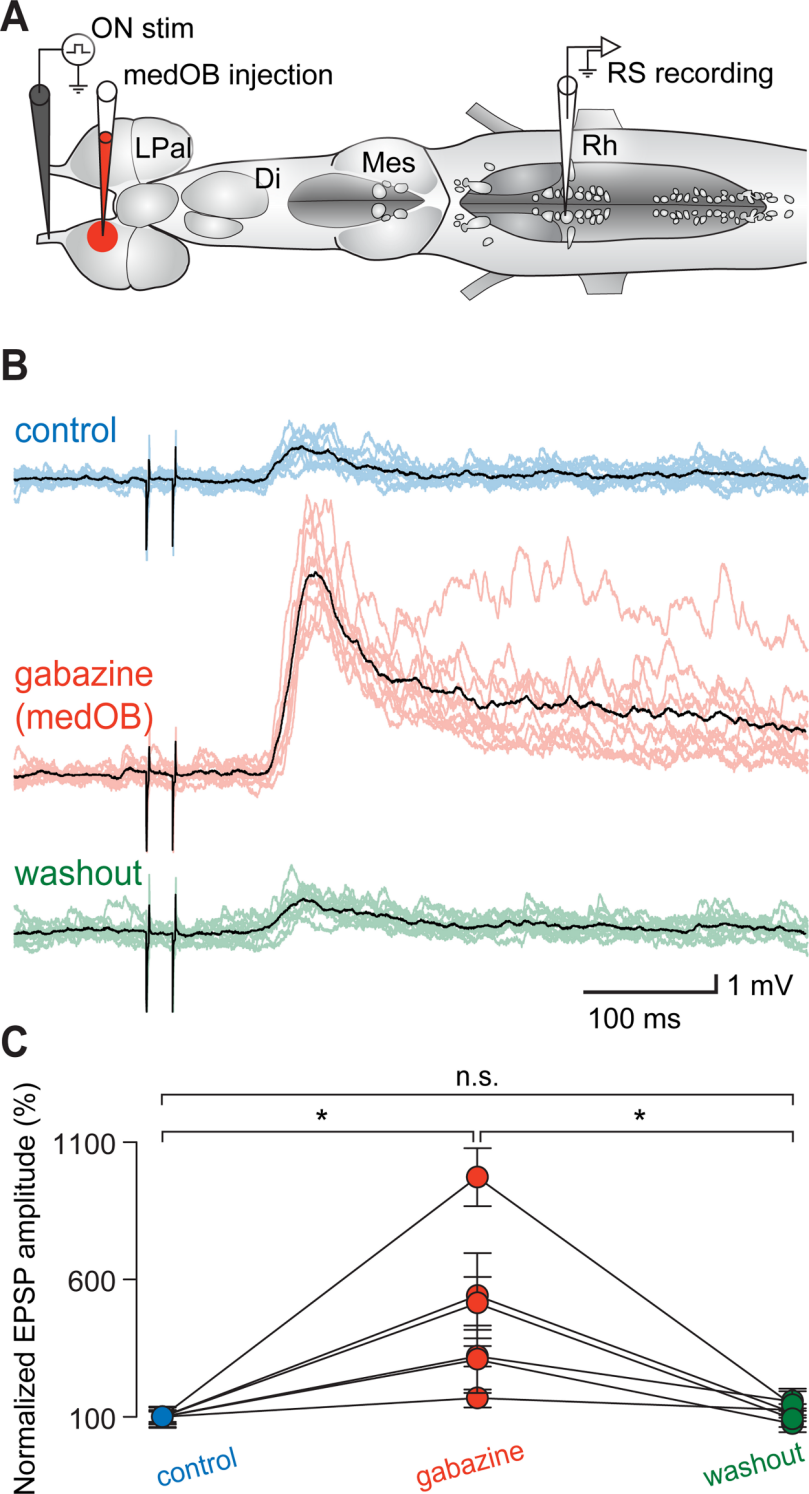
Injection of gabazine into the medOB increases RS neuron responses to ON stimulation. (A) Schematic representation of the brain preparation illustrating the stimulation, injection, and recording sites. (B) Responses of RS neurons to electrical stimulation of the ON (5–15 μA). A local injection of gabazine (0.1 mM) in the medOB increased the RS neuron responses to ON stimulation. Each black trace is a mean of 10 individual responses (colored traces). (C) Univariate scatterplot showing the normalized (as a percentage of control) EPSP amplitude in control, gabazine, and washout conditions for all animals. An asterisk (*) indicates a statistically significant difference at the level *p* < 0.05, while n.s. indicates the absence of statistically significant difference. The numerical values underlying this figure can be found in S1 Data. Di, diencephalon; EPSP, excitatory postsynaptic potential; LPal, lateral pallium; medOB, medial part of the olfactory bulb; Mes, mesencephalon; ON, olfactory nerve; Rh, rhombencephalon; RS, reticulospinal.

**Fig 3:**
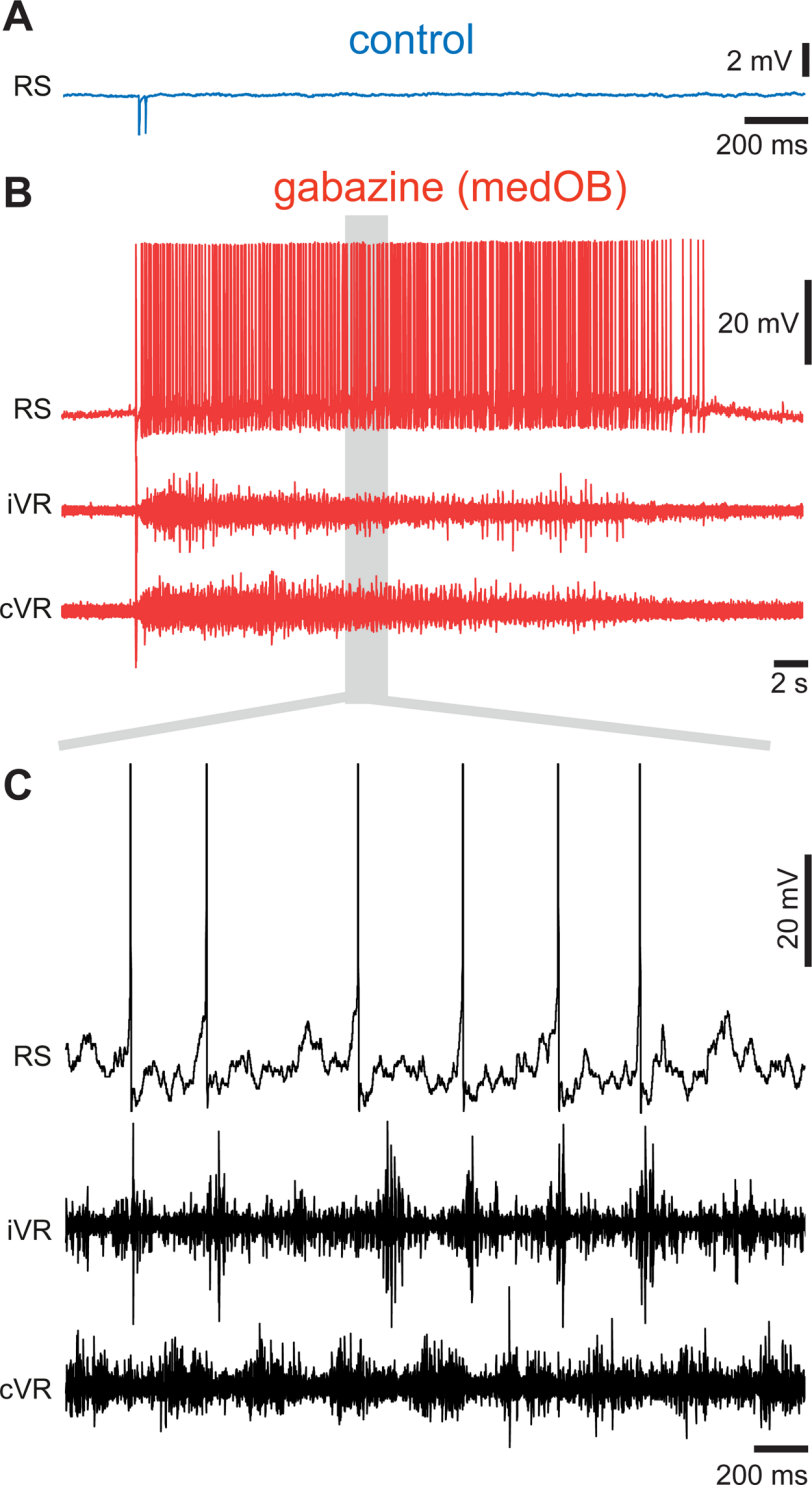
Stimulation of the ON after a gabazine injection into the medOB can induce fictive swimming. (A, B) Responses of a RS neuron to electrical stimulation of the ON (10 μA) before (A) and after (B, top trace) a local injection of gabazine (1 mM) in the medOB. Bottom traces in (B) show the neural activity in the iVR and cVR. (C) Details from the gray area in (B) showing fictive locomotion that is characterized by rhythmic alternating ventral root discharges on the iVR and cVR sides. The data and statistics on trials can be found in S1 Data. cVR, contralateral ventral root; iVR, ipsilateral ventral root; medOB, medial part of the olfactory bulb; ON, olfactory nerve; RS, reticulospinal.

To investigate the physiological role of the GABAergic circuitry in the OB, local microinjections of the GABA_A_ receptor antagonist, gabazine, were made into restricted areas of the OB, while stimulating the olfactory nerve (ON) and intracellularly recording from RS neurons on the same side of the brain. Gabazine injections (0.1 mM, 1.4 ± 1.6 nL) in the medOB (*n* = 60 synaptic responses; *n* = 6 neurons; *n* = 6 larval animals; Fig 2) were found to amplify synaptic responses of RS neurons to electrical stimulation of the ON (amplitude increase of 372.2 ± 277.5%; *p* < 0.05; no statistical differences between control and washout; Fig 2B and 2C). RS neurons from all four reticular nuclei (mesencephalic reticular nucleus and anterior, middle, and posterior rhombencephalic reticular nuclei) responded similarly as shown by calcium imaging experiments (*n* = 362 neurons; *n* = 6 adult animals, S3 Fig). Extracellular recordings of the OB further showed that responses of OB neurons to ON stimulation were greatly increased under gabazine (*n* = 60 responses; *n* = 6 animals, S4 Fig), thus corroborating our previous findings. In addition to increasing the responses of RS cells, stimulation of the ON after gabazine injection in the medOB even induced motor discharges in the ventral roots. The neural activity consisted of rhythmic discharges alternating on both sides, a hallmark of fictive swimming (in 58.1% of trials; *n* = 36 locomotor bouts out of 62 trials for gabazine versus 0 out of 78 for control; *n* = 9: three adult animals and six larval animals, Fig 3 and S1 Data).

**Fig 4:**
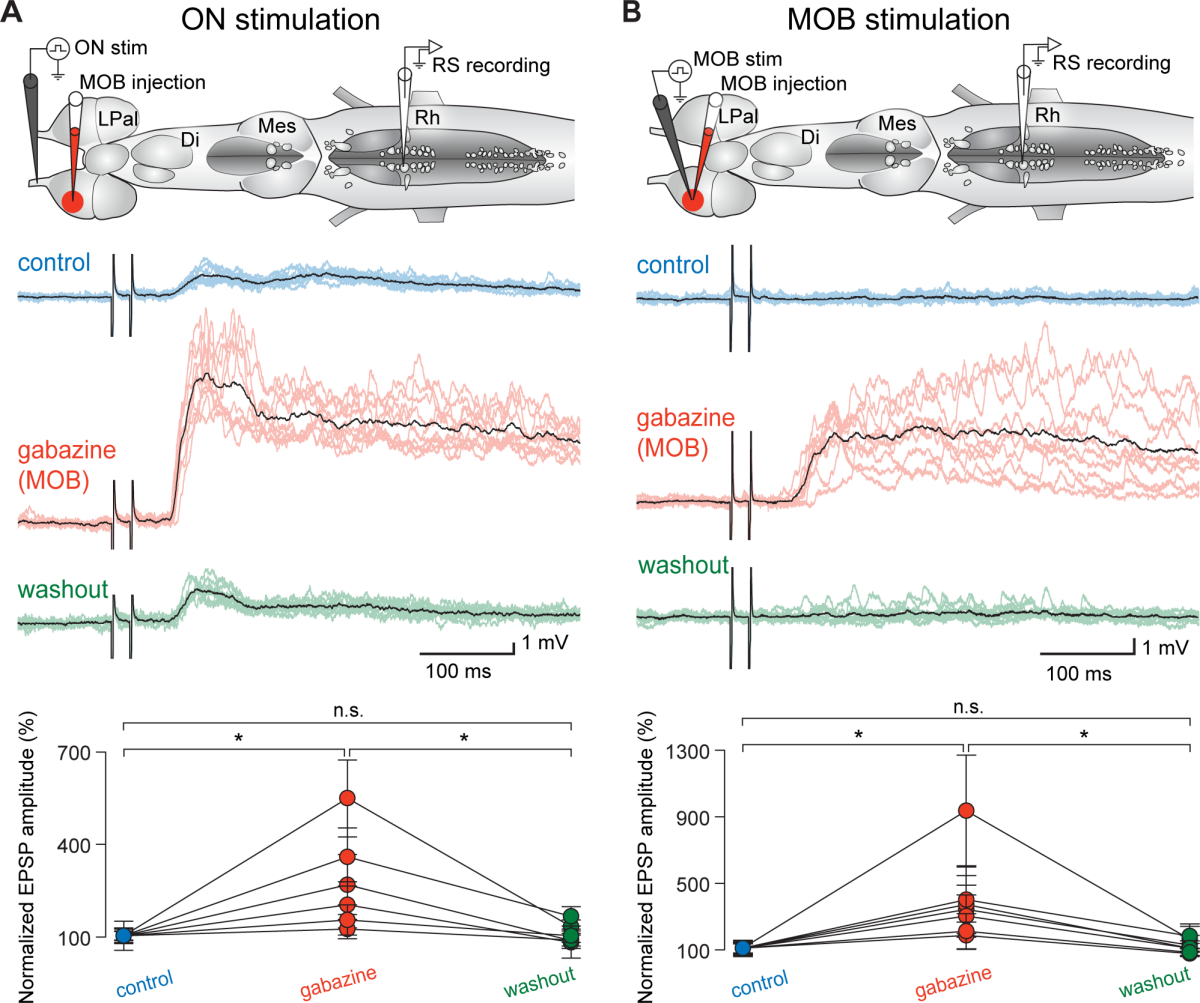
Injection of gabazine into the MOB also increases RS neuron responses to olfactory stimulation. (A) Top: schematic illustration of the brain showing stimulation, injection, and recording sites. Middle: responses of RS neurons to the electrical stimulation of the ON. A local injection of gabazine (0.1 mM) in the MOB increased the RS neuron responses to ON stimulation (5–10 μA). Each black trace is the mean of 10 individual responses (colored traces). Bottom: univariate scatterplot showing the normalized (as a percentage of control) EPSP amplitude in control, gabazine, and washout conditions for all animals. (B) Top: schematic illustration of the brain shows stimulation, injection, and recording sites. Middle: responses of RS neurons to the electrical stimulation of the MOB. Under control conditions, MOB stimulation did not induce responses in RS neurons. After a local injection of gabazine (0.1 mM) in the MOB, RS neurons responded to MOB stimulation (5–20 μA). Each black trace is the mean of 10 individual responses (colored traces). Bottom: univariate scatterplot showing the normalized (as a percentage of control) EPSP amplitude in control, gabazine, and washout conditions for all animals. An asterisk (*) indicates a statistically significant difference at the level *p* < 0.05, while n.s. indicates the absence of a statistically significant difference. The numerical values underlying this figure can be found in S1 Data. Di, diencephalon; EPSP, excitatory postsynaptic potential; LPal, lateral pallium; Mes, mesencephalon; MOB, main olfactory bulb; ON, olfactory nerve; Rh, rhombencephalon; RS, reticulospinal.

**Fig 5:**
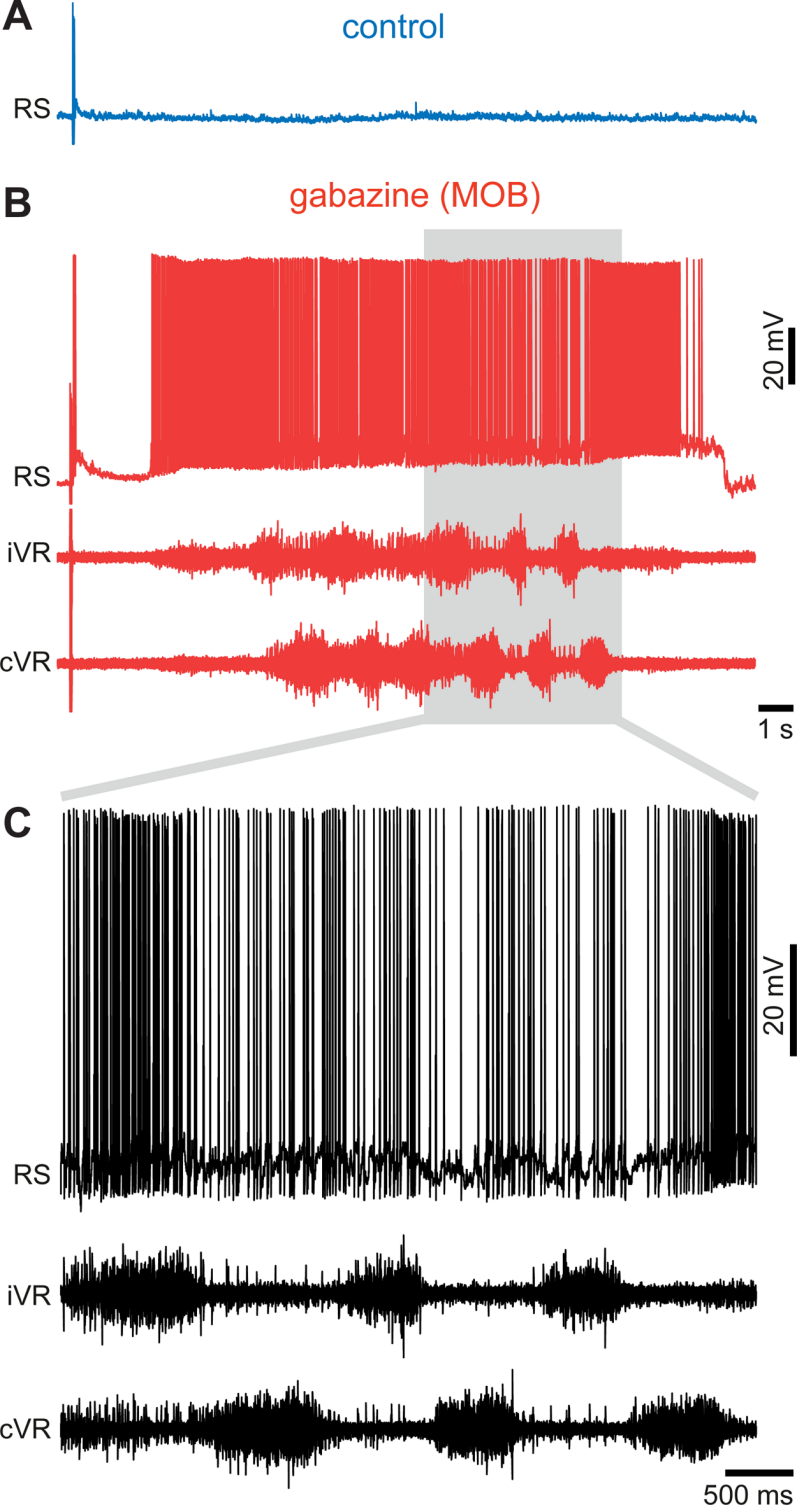
Stimulation of the MOB after an injection of gabazine into the MOB can induce fictive swimming. (A, B) Responses of a RS neuron to electrical stimulation of the MOB (30 μA) before (A) and after (B, top trace) a gabazine (1 mM) injection into the MOB. Bottom traces in (B) show the neural activity in the iVR and cVR. (C) Detail from the gray area in (B) showing that the neural activity corresponds to fictive locomotion that is characterized by rhythmic alternating ventral root discharges on the iVR and cVR sides. The data and statistics on trials can be found in S1 Data. cVR, contralateral ventral root; iVR, ipsilateral ventral root; MOB, main olfactory bulb; RS, reticulospinal.

Because the density of GABAergic processes seemed relatively similar in the medOB and the MOB, we hypothesized that the neural activity in the MOB could be modulated by GABA, as observed for the medOB. To test this hypothesis, the effect of gabazine injections in the MOB on RS cell responses was examined. As shown for the medOB, gabazine injections into the MOB (0.1 mM, 1.8 ± 2.0 nL) enhanced the RS neuron responses to ON stimulations (*n* = 60 synaptic responses; *n* = 6 neurons; *n* = 6 larval animals; amplitude increase of 174.4 ± 167.0%, *p* < 0.05; no statistical differences between control and washout; Fig 4A). However, stimulation of the ON does not activate MOB neurons specifically, as it also activates medOB neurons. To rule out any involvement of the medOB in the increased RS responses after MOB gabazine injections, the effect of an electrical stimulation of the MOB with a gabazine injection (0.1 mM, 2.9 ± 1.1 nL) in the MOB was tested. Under control conditions, MOB stimulation did not induce responses in RS neurons. However, after a gabazine injection in the MOB, electrical stimulation of the MOB elicited responses in RS cells (*n* = 70 synaptic responses; *n* = 7 neurons; *n* = 7 larval animals; amplitude increase of 286.3 ± 296.8%; *p* < 0.05; no statistical differences between control and washout; Fig 4B). As a further control, electrical stimulation of the MOB under gabazine elicited significant responses in RS cells, even when the medOB had been surgically resected (*n* = 5 larval animals, S5 Fig). Furthermore, recordings of the ventral roots of the spinal cord showed that electrical stimulation of the MOB after a gabazine injection in the MOB can induce fictive swimming (in 65.1% of trials; 54 locomotor bouts out of 83 trials for gabazine versus 0 out of 105 for control; *n* = 9 larval animals, Fig 5 and S1 Data). Taken together, these findings suggest the presence of a previously unknown pathway linking the MOB to RS cells that seems to be under a strong tonic GABAergic inhibitory control.

### A lateral olfactomotor pathway

**Fig 6:**
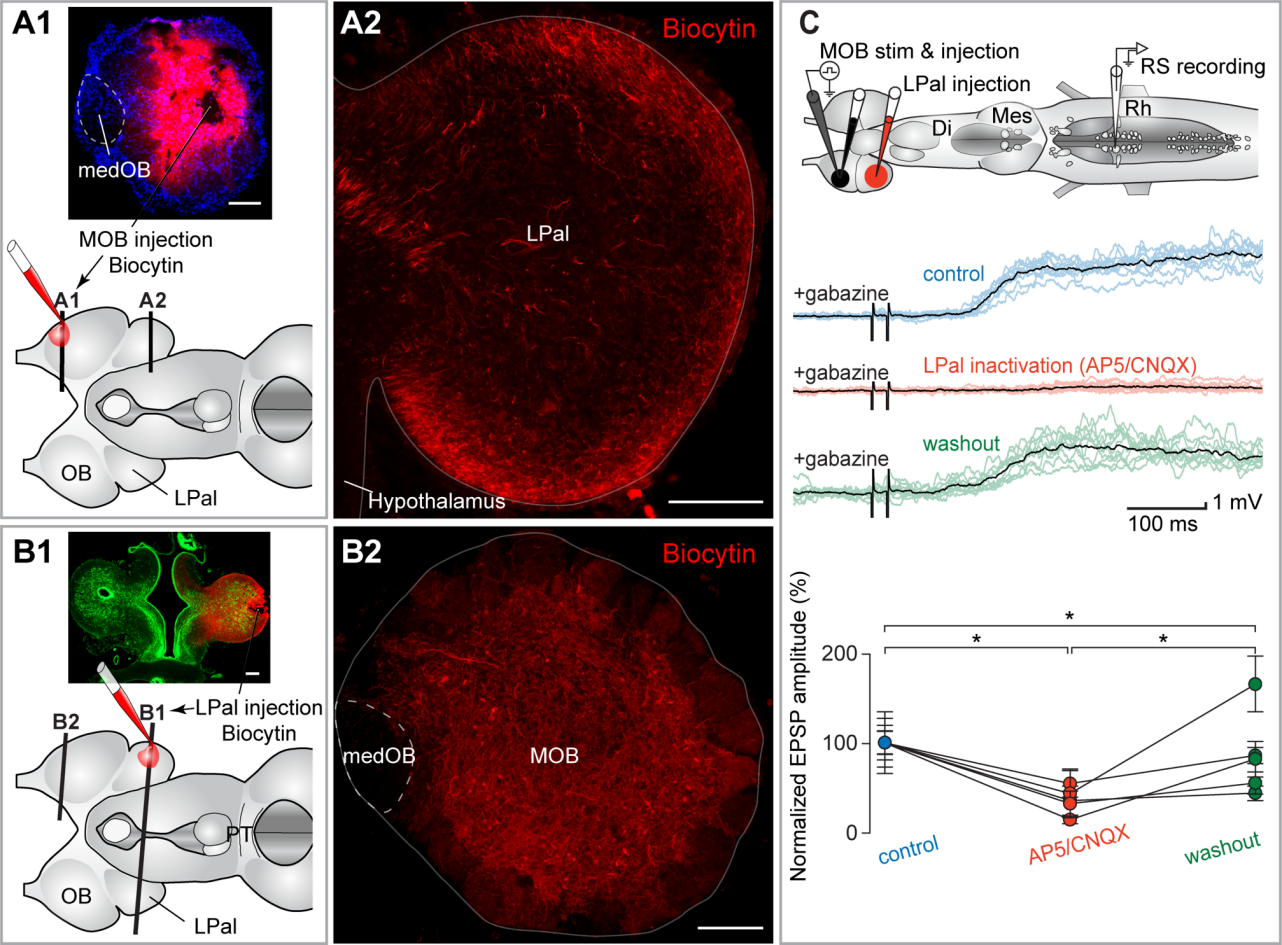
The MOB inputs to RS neurons are relayed in the LPal. (A1) Schematic representation of the brain illustrating the injection site in the MOB and the level of the cross sections shown in A1 and A2. (A2) Numerous fibers were labeled anterogradely in the LPal after a biocytin injection that included a large portion of the MOB but spared the medOB (see A1). (B1) Schematic illustration of the brain showing the injection site in the LPal and the level of the cross sections shown in B1 and B2. (B2) Projection neurons in the MOB were retrogradely labeled after an injection of biocytin in the LPal. These neurons were mainly located in the external portion of the ICL, close to the glomeruli, as opposed to the GABA-containing neurons that were more internally located in the ICL (Fig 1A and S1 Fig). The level of the injection in the LPal in B1 is similar to the level of the section illustrated in A2. Note that the medOB, which contains neurons that project directly to the PT, does not contain neurons projecting to the LPal. (C) Top: schematic illustration of the brain showing stimulation, injection, and recording sites. Middle: responses of RS neurons to the electrical stimulation (15 μA) of the MOB after injecting gabazine (0.1 mM) in the MOB. A local injection of glutamate receptor antagonists AP5/CNQX (0.5 mM/1 mM) in the LPal abolished the RS neuron responses (red traces). Each black trace is the mean of 10 individual responses (colored traces). Bottom: univariate scatterplot showing the normalized (as a percentage of control) EPSP amplitude in control, AP5/CNQX, and washout conditions for all animals. An asterisk (*) indicates a statistically significant difference at the level *p* < 0.05, while n.s. indicates the absence of statistically significant difference. The numerical values underlying this figure can be found in S1 Data. Scale bars in A, B = 200 μm. AP5, 2-amino-5-phosphonopentanoic acid; CNQX, 6-cyano-7-nitroquinoxaline-2,3-dione; Di, diencephalon; EPSP, excitatory postsynaptic potential; ICL, internal cell layer; LPal, lateral pallium; medOB, medial part of the olfactory bulb; Mes, mesencephalon; MOB, main olfactory bulb; PT, posterior tuberculum; Rh, rhombencephalon; RS, reticulospinal; stim, stimulation.

We investigated the spatial organization of projections from the MOB that would eventually reach the RS neurons. We injected the axonal tracer biocytin in the MOB (*n* = 13 adult animals, Fig 6A1) and found ipsilateral axonal projections to the lateral pallium (LPal), medial pallium, dorsal pallium, striatum, dorsomedial telencephalic neuropil, and habenula. Contralateral projections were found to the OB, dorsomedial telencephalic neuropil, striatum, and LPal. The MOB injections did not label any fibers in the PT. Similar olfactory projections from the OB have been reported in other species of lampreys [46,47], but the selective contribution from the medOB or the MOB was not investigated in these earlier studies. The LPal appears to be a major target of neurons in the MOB, judging by the numerous labeled fibers seen to enter this region. The fibers densely filled the outermost layer covering the entire rostro-caudal extent of the LPal (Fig 6A2). Many fibers were also seen in the more central layers of the LPal, where the neuronal cell bodies of that structure are located. Tracer injections in the LPal (*n* = 9 adult animals, Fig 6B1) retrogradely labeled many neurons in the MOB without ever labeling cell bodies in the medOB (Fig 6B2). The retrolabeled neurons in the MOB were found close to the glomeruli, but were almost never seen inside them.

Physiological experiments were then carried out to characterize the effect of the pharmacological inactivation of the LPal on the responses of RS cells to the electrical stimulation of the MOB. Based on the results reported in Fig 4B, these experiments were carried out after removing the local GABAergic inhibition with a gabazine microinjection into the MOB (0.1 mM, 0.9 ± 1.0 nL, just prior each stimulation). An injection of glutamate receptor antagonists (2-amino-5-phosphonopentanoic acid [AP5]: 0.5 mM, 6-cyano-7-nitroquinoxaline-2,3-dione [CNQX]: 1 mM, 5.2 ± 0.8 nL) in the LPal strongly decreased the RS neuron responses (amplitude decrease of 64.8 ± 21.3%; *p* < 0.05), thus confirming the role of the LPal in relaying glutamatergic outputs from the MOB to locomotor control centers (*n* = 50 synaptic responses; *n* = 5 neurons; *n* = 5 larval animals, Fig 6C).

**Fig 7:**
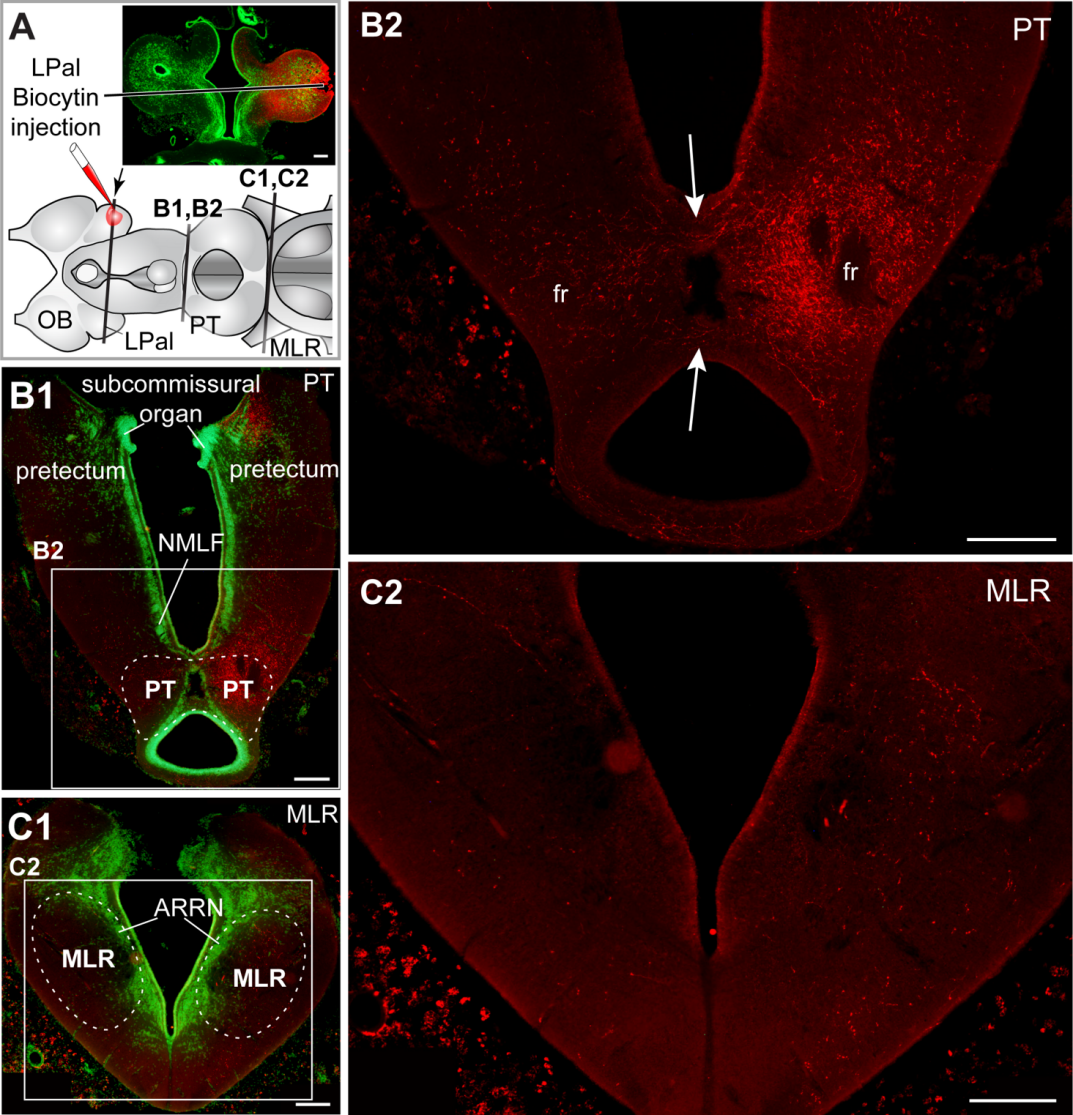
The LPal projects to the PT and MLR. (A) Schematic illustration of the brain showing the injection site in the LPal and levels of the cross sections shown in A–C. (B) Many descending fibers from the LPal (red) reach the PT. (B1) Cross section at the level of the PT with a superimposed fluorescent Nissl stain (green). (B2) Some fibers are seen crossing to the contralateral side (arrows) at this level. Note that the Nissl stain is not illustrated here. (C) A number of descending fibers from the LPal (red) reach the MLR. (C1) Cross section at the level of the MLR with a superimposed fluorescent Nissl stain (green). (C2) Most fibers from the LPal innervate the ipsilateral MLR (right), although a few fibers were also seen contralaterally (left). Note that the Nissl stain is not illustrated here. B1 and C1 are photomontages of higher-power photomicrographs. All results come from the same animal as in Fig 6B. All scale bars = 200 μm. ARRN, anterior rhombencephalic reticular nucleus; fr, fasciculus retroflexus; LPal, lateral pallium; MLR, mesencephalic locomotor region; NMLF, nucleus of the medial longitudinal fasciculus; OB, olfactory bulb; PT, posterior tuberculum.

Biocytin was injected in the LPal to examine its descending projections. Emphasis was placed on regions known to be involved in the medial olfactomotor pathway, such as the PT and the MLR (*n* = 5 adult animals, Fig 7). Numerous fibers terminated in the PT, predominantly on the ipsilateral side (Fig 7B), with fibers crossing locally to the contralateral side (arrows in Fig 7B2). At levels immediately caudal to the PT, in the rostral mesencephalon, the number of descending fibers decreased sharply. Only a few labeled fibers continued to the level of the MLR (Fig 7C), where many appeared to terminate (Fig 7C2). More caudal levels were not investigated in the present study, but it is not excluded that some fibers continued down more caudally [48].

**Fig 8:**
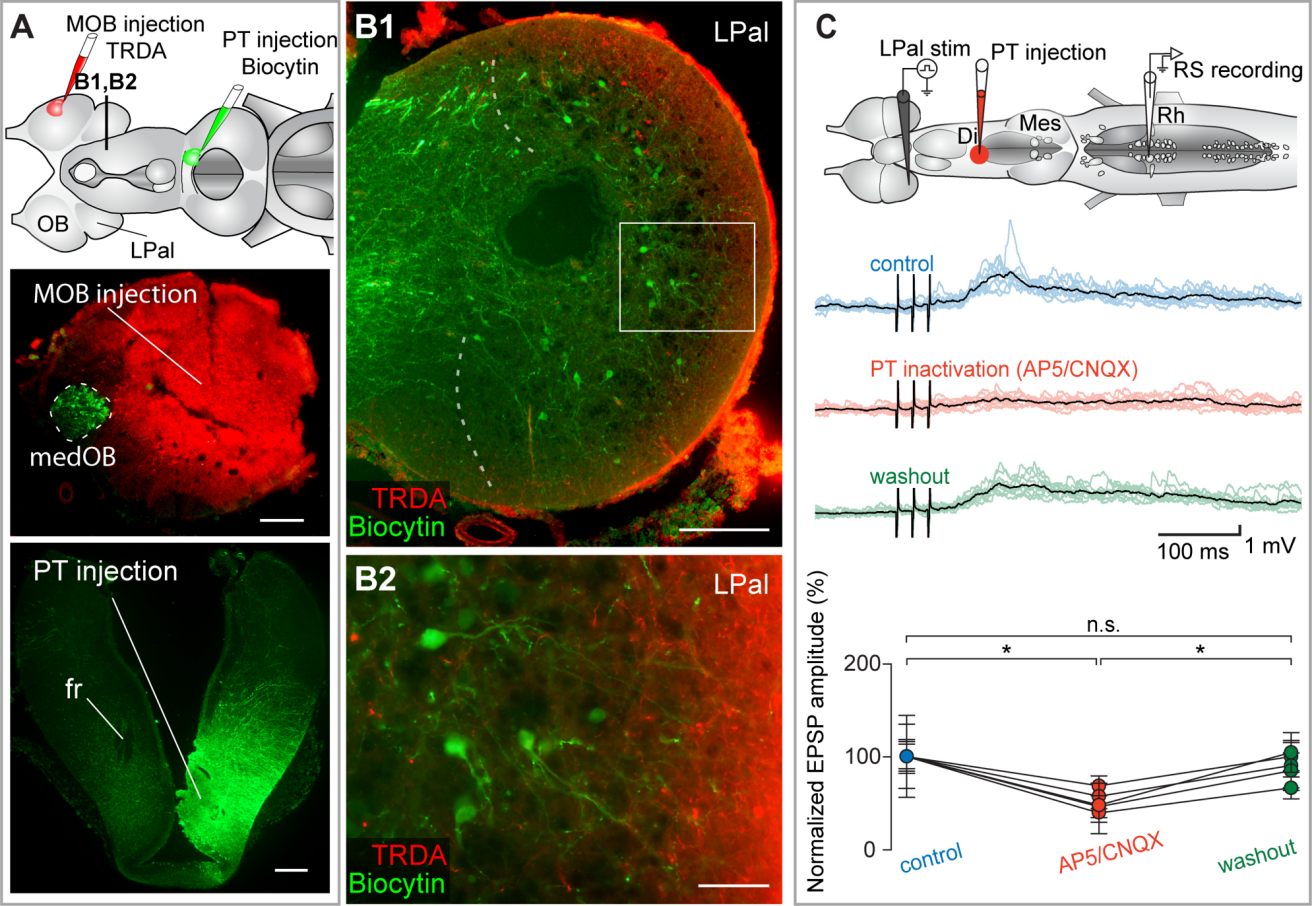
The LPal inputs to RS neurons are relayed in the PT. (A) Top: schematic representation of the brain illustrating the tracer injection sites and the level of the cross section shown in B. Bottom: the injection sites are illustrated on cross sections. Many projection neurons (green), found only in the medOB, are retrogradely labeled from the PT injection. (B1) Many neurons (in green) are retrogradely labeled in the LPal after a tracer injection in the PT. Descending fibers labeled from an MOB injection are superimposed (red). The medial border of the LPal is indicated by curved dashed lines. (B2) High magnification of the boxed area in B1 showing dendrites of neurons projecting to the PT (green) extending towards the descending fibers from the MOB (red). (C) Top: schematic illustration of the brain showing stimulation, injection, and recording sites. Middle: RS neuron responses to electrical stimulation (15–20 μA) of the LPal. A local injection of the glutamate receptor antagonists AP5/CNQX (0.5 mM/1 mM) in the PT (see schematized brain, upper panel) strongly decreased the RS neuron responses. Each black trace is a mean of 10 individual responses (colored traces). Bottom: univariate scatterplot showing the normalized (as a percentage of control) EPSP amplitude in control, AP5/CNQX, and washout conditions for all animals. An asterisk (*) indicates a statistically significant difference at the level *p* < 0.05, while n.s. indicates the absence of statistically significant difference. The numerical values underlying this figure can be found in S1 Data. Scale bars in A, B1 = 200 μm; scale bar in B2 = 50 μm. AP5, 2-amino-5-phosphonopentanoic acid; CNQX, 6-cyano-7-nitroquinoxaline-2,3-dione; Di, diencephalon; EPSP, excitatory postsynaptic potential; fr, fasciculus retroflexus; LPal, lateral pallium; medOB, medial part of the olfactory bulb; Mes, mesencephalon; MOB, main olfactory bulb; PT, posterior tuberculum; Rh, rhombencephalon; RS, reticulospinal; stim, stimulation; TRDA, Texas Red-conjugated dextran amine.

**Fig 9:**
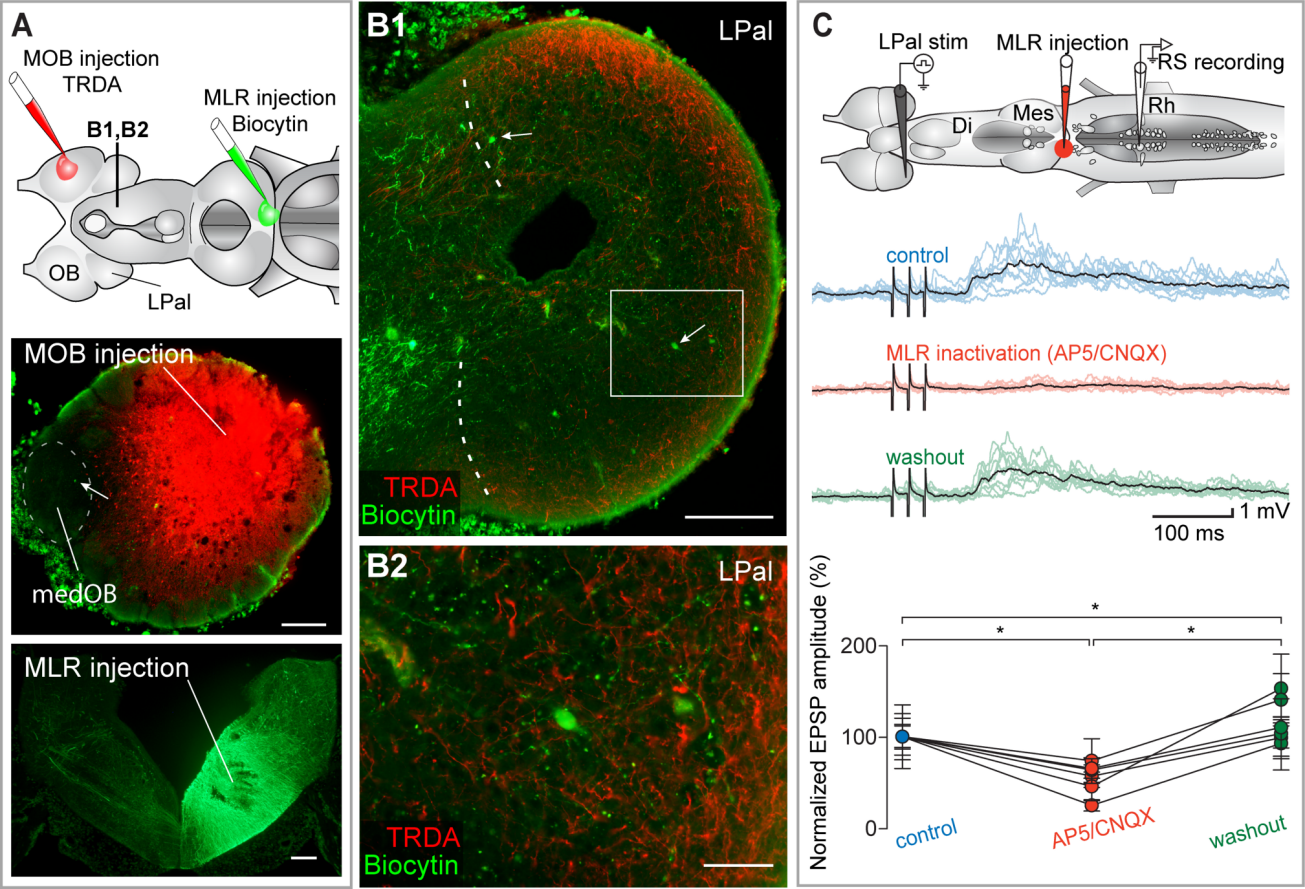
The LPal inputs to RS neurons are also relayed in the MLR. (A) Top: schematic illustration of the brain showing the tracer injection sites and the level of the cross section shown in B. Bottom: the injection sites are illustrated on cross sections. A few medOB projection neurons are consistently retrogradely labeled by an MLR injection (one neuron here shown by an arrow in the photomicrograph of the OB). (B1) Neurons (green, two here, arrows) are retrogradely labeled in the LPal after a tracer injection in the MLR. Descending fibers labeled from a MOB injection are superimposed (red). The medial border of the LPal is indicated by curved dashed lines. (B2) High magnification of the boxed area in B1 showing neurons projecting to the MLR (green) in proximity to descending fibers from the MOB (red). (C) Top: schematic illustration of the brain showing stimulation, injection, and recording sites. Middle: RS neuron responses to the electrical stimulation (15–20 μA) of the LPal. A local injection of the glutamate receptor antagonists AP5/CNQX in the MLR (see schematized brain, upper panel) strongly decreased the RS neuron responses. Each black trace is a mean of 10 individual responses (colored traces). Bottom: univariate scatterplot showing the normalized (as a percentage of control) EPSP amplitude in control, AP5/CNQX, and washout conditions for all animals. An asterisk (*) indicates a statistically significant difference at the level *p* < 0.05, while n.s. indicates the absence of statistically significant difference. The numerical values underlying this figure can be found in S1 Data. Scale bars in A, B1 = 200 μm; scale bar in B2 = 50 μm. AP5, 2-amino-5-phosphonopentanoic acid; CNQX, 6-cyano-7-nitroquinoxaline-2,3-dione; Di, diencephalon; EPSP, excitatory postsynaptic potential; LPal, lateral pallium; medOB, medial part of the olfactory bulb; Mes, mesencephalon; MLR, mesencephalic locomotor region; MOB, main olfactory bulb; OB, olfactory bulb; Rh, rhombencephalon; RS, reticulospinal; stim, stimulation; TRDA, Texas Red-conjugated dextran amine.

Tracing experiments were carried out to further characterize the population of LPal neurons projecting to the PT and the MLR. The organization and anatomical boundaries of the LPal in lamprey are still debated [47,49–56]. In the present study, we followed the nomenclature of Northcutt and Puzdrowski [47] and Pombal and Puelles [54]. The part of the brain that was considered to be the LPal in the present study is illustrated in S6 Fig. In this series of experiments, a solution containing Texas Red-conjugated dextran amine (TRDA) was injected in the MOB to label olfactory projections from the OB and a solution containing biocytin was injected in the PT (*n* = 7 adult animals and 1 larval animal, Fig 8A and S7 Fig) or the MLR (*n* = 4 adult animals and 1 larval animal, Fig 9A and S7 Fig) to retrogradely label neurons projecting to the PT or MLR. Typical results are shown in Figs 8 and 9 and S7 Fig. Labeled cell bodies were distributed uniformly in all regions of the LPal, dorsal, ventral, rostral and caudal, when injections were made in the PT (Fig 8A and 8B) or the MLR (Fig 9A and 9B). The dendrites of cells often extended radially towards the outermost layer of the LPal, where secondary olfactory fibers, labeled from the MOB, are located (Fig 8B and Fig 9B). These results show that fibers originating in the MOB came in proximity with LPal neurons projecting to both the PT and the MLR, suggesting that the LPal is a relay for MOB inputs to the PT and MLR.

Electrophysiological experiments were then conducted to examine the effect of deactivating the PT and the MLR on the RS neuron responses to the electrical stimulation of the LPal (Fig 8C and Fig 9C, respectively). Glutamate antagonists were locally injected in either the PT (AP5: 0.5 mM, CNQX: 1 mM, 1.1 ± 1.2 nL, Fig 8C) or the MLR (AP5: 0.5 mM, CNQX: 1 mM, 3.6 ± 2.5 nL, Fig 9C), and the RS neuron responses were markedly decreased (PT: amplitude decrease of 48.7 ± 19.7%; *p* < 0.05; no statistical differences between control and washout; *n* = 50 synaptic responses; *n* = 5 neurons; *n* = 5 larval animals; MLR: amplitude decrease of 45.3 ± 21.7%; *p* < 0.05; *n* = 60 synaptic responses; *n* = 6 neurons; *n* = 6 larval animals). Taken together with our previous findings, these results show that glutamatergic olfactory outputs from the MOB are relayed via the LPal to the PT and to the MLR before reaching RS cells.

**Fig 10:**
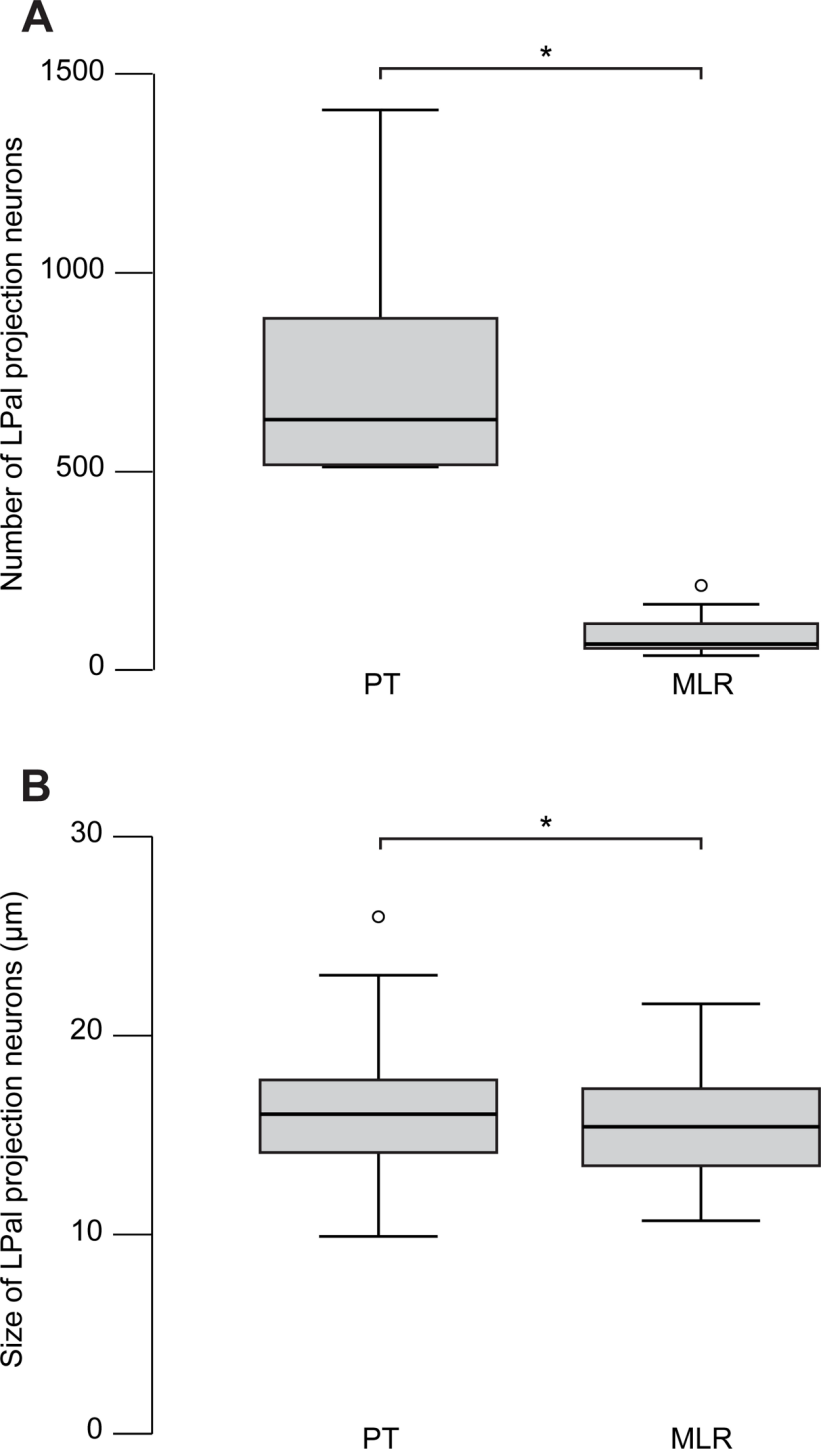
Number and size of LPal neurons projecting to the PT and MLR. (A) Box plot showing the number of retrogradely labeled neurons in the LPal after injections of axonal tracer in the PT or in the MLR. (B) Box plot showing the size of retrogradely labeled neurons in the LPal after injections of axonal tracer in the PT or in the MLR. An asterisk (*) indicates a statistically significant difference at the level *p* < 0.05. The numerical values underlying this figure can be found in S1 Data. LPal, lateral pallium; MLR, mesencephalic locomotor region; PT, posterior tuberculum.

The relative importance of the projection from the LPal to the PT or to the MLR was examined by counting retrogradely labeled cells in the LPal after an injection of a fluorescent tracer in the PT or the MLR. Bilateral biocytin injections in the PT (*n* = 6 adult animals) followed by the analysis of 10 LPals revealed that, on average, 751 ± 283 LPal neurons (per LPal) projected to the PT (Fig 10A). Bilateral biocytin injections in the MLR (*n* = 5 adult animals) followed by the analysis of eight LPals revealed an average of 93 ± 62 neurons (per LPal) in these animals (Fig 10A). The size of LPal neurons projecting to the PT and MLR was measured along their long axis. LPal PT- and MLR-projecting neurons measured on average 16.3 ± 3.0 μm (*n* = 90 cells from a subset of three animals, Fig 10B) and 15.4 ± 2.4 μm (*n* = 90 cells from a subset of three animals, Fig 10B), respectively. Interestingly, a few medOB neurons were systematically labeled after an MLR tracer injection (Fig 9A), thus demonstrating a direct projection from the medOB to the MLR.

**Fig 11:**
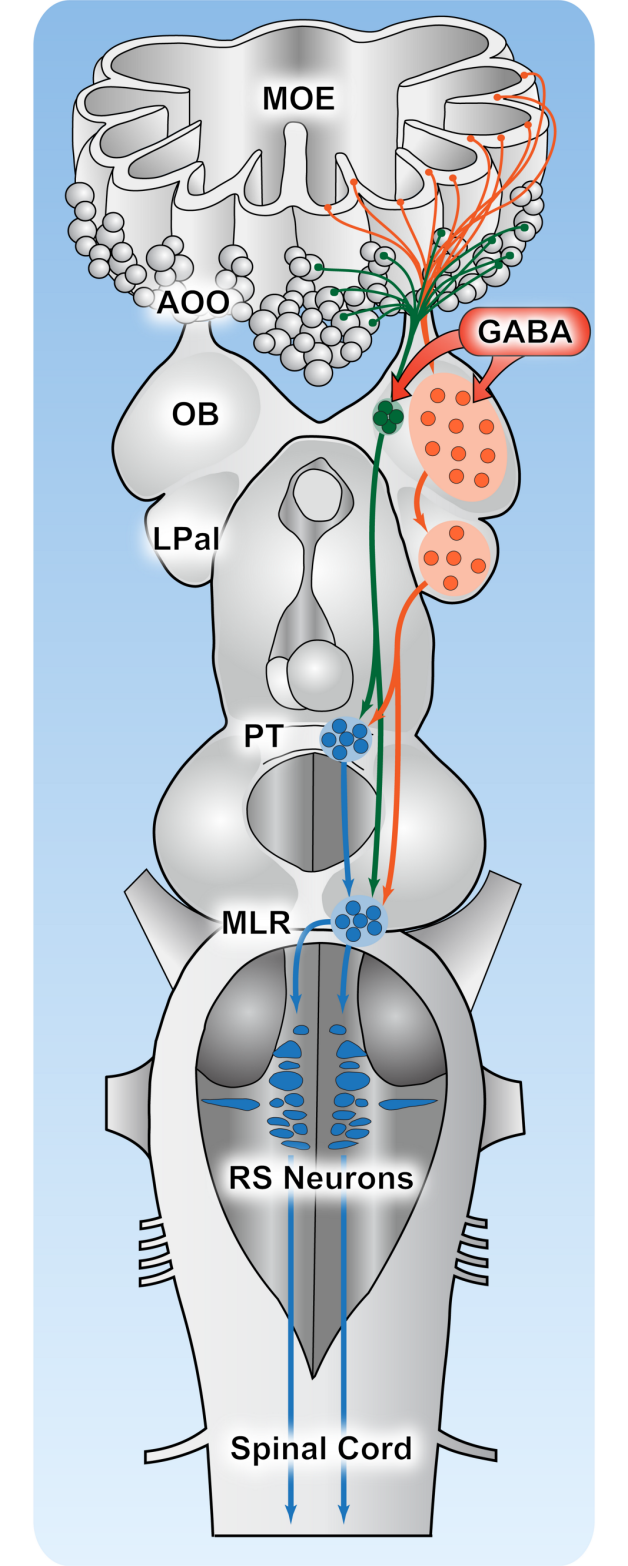
Two olfactomotor pathways in lampreys. Schematic representation of the brain illustrating a medial (green) and a lateral (orange) olfactomotor pathways. Both pathways act on the downstream locomotor control circuitry (blue) and thus contribute to olfactory-induced locomotor behaviors in lampreys. GABA (red) modulates both pathways at the level of the olfactory bulb. AOO, accessory olfactory organ; LPal, lateral pallium; MLR, mesencephalic locomotor region; MOE, main olfactory epithelium; OB, olfactory bulb; PT, posterior tuberculum; RS, reticulospinal.

The lateral olfactomotor pathway (orange pathway in Fig 11) may contribute significantly to the motor responses of lampreys to olfactory cues in their environment, in parallel to the previously described medial olfactomotor pathway (green pathway in Fig 11).

## Discussion

The transformation of sensory inputs into motor outputs is critical for the survival and reproduction of animals. As such, it represents an important part of their behavioral repertoire. While phylogenetically old nervous systems are predominantly dedicated to sensorimotor behaviors, the underlying neural pathways have been characterized in only a few cases. Recent studies in invertebrates have shed light on the neural substrate underlying some sensorimotor behaviors [57–59]. In contrast, very limited information has been collected in relation to the mechanisms of the transformation of sensory inputs into a locomotor output in vertebrates. For instance, it is well known that most animals (including humans) display odor-guided behaviors. However, the neural substrate and mechanisms associated with these behaviors have remained poorly understood.

In this study, we show that a GABAergic circuitry in the OB modulates a hardwired sensorimotor pathway. This is, to the best of our knowledge, the first report of the role of the OB GABAergic circuitry in modulating a motor behavior in any vertebrate species. Moreover, we identified a lateral olfactomotor pathway originating in the MOB and reaching locomotor control centers via a relay in the LPal. Together with our previous discovery of the medial olfactomotor pathway [12], these findings support the existence of two olfactomotor pathways in one of the most basal extant vertebrate species, the sea lamprey. As basal vertebrates, lampreys share a common brain “bauplan” with jawed vertebrates, including modern-day mammals. Therefore, knowledge gained from lamprey circuits and mechanisms provides insight into fundamental principles of vertebrate brain organization and function.

### Modulation of the olfactomotor transmission

Lampreys, like many other animal species, display sex- and life stage–specific olfactory-induced motor behaviors [60–63]. The neural mechanisms accounting for the behavioral variability associated with a specific neural pathway within a species are largely unknown. However, the long-standing hypothesis that it was due to fundamental differences in brain wiring is now being challenged (reviewed in [64]). Indeed, only very subtle sex-specific differences have been found in the structure and circuitry of the brain in mammals [65–68]. Likewise, we have shown in a previous study that a hardwired olfactomotor pathway is present in both sexes at all life stages in the sea lamprey [12]. For this reason, we hypothesized in the present study that modulatory mechanisms acting on this pathway could play a role in the variability of the behavioral responses of lampreys to olfactory cues.

The OB is the first relay of the olfactomotor pathway. As such, it interfaces sensory afferents with motor control centers and it is ideally located to modulate olfactory-induced motor responses in lampreys. It has been proposed that the main function of the OB in vertebrates is the filtering and transmission of olfactory inputs [69]. Studies in mammals and turtles have shown that the sensory inputs to the OB are modulated both at presynaptic and postsynaptic levels by two classes of local GABAergic interneurons: periglomerular and granule cells [69]. Periglomerular cells inhibit glutamate release from primary olfactory axon terminals via a GABA_B_-mediated mechanism [70–73]. On the other hand, granule cells inhibit projection neurons via a GABA_A_-mediated mechanism [74–78]. Despite a rather good understanding of the cellular mechanisms responsible for the modulation of olfactory inputs, little is known about their overall effect on the OB output and, ultimately, on the resulting behavior. Using an in vitro isolated preparation of lamprey CNS, we provide the first evidence linking cellular GABAergic modulatory mechanisms in the OB to the activation of a sensorimotor pathway producing locomotor behavior.

We showed that the lamprey OB anatomical organization is very similar to that of other vertebrates, regarding its GABAergic circuitry. Our material confirms previous work showing that the lamprey OB contains numerous GABAergic neurons of different morphological types [43]. The morphology and location of the GABAergic neurons suggest that they are mainly granule cells [43,45], but not excluding possible periglomerular cells [43,51]. We also showed that both medOB and MOB glomeruli are densely innervated with GABAergic processes. These GABAergic processes are in close proximity to both primary olfactory axon terminals and dendrites or somata of OB projection neurons; this suggests possible pre- or postsynaptic contacts (S2 Fig). We have not formally identified types (i.e., axons versus dendrites) and origin (i.e., intrinsic versus extrinsic) of the GABAergic processes. The abundant GABAergic cell bodies labeled in the OB suggest that they may be of intrinsic (OB) origin (i.e., granule cells or periglomerular cells), as seen in other vertebrate species [39–43]. The lamprey granule cells are axonless [45], as in other vertebrate species. These processes are thus likely to be dendrites of granule cells or dendrites and axons of periglomerular cells, but some of these processes could be axons originating from neurons located in other parts of the brain. In mammals, most neuromodulatory inputs to the OB originate from the locus coeruleus (noradrenergic inputs), the nucleus of the diagonal band of Broca (cholinergic inputs), and the midbrain raphe (serotoninergic) (reviewed in [69,79,80]). However, some cells located in the nucleus of the diagonal band of Broca are GABAergic and project to the OB [81,82].

We now show that injection of the GABA_A_ receptor antagonist, gabazine, in the OB potentiates RS cell responses to ON or OB stimulation, thus suggesting an enhancement of the olfactomotor transmission. In the case of OB (MOB) stimulation, however, we cannot completely exclude that electrical stimulation of the MOB might recruit not only projection neurons but also local GABAergic interneurons, and that in such a case, an injection of gabazine might block the effect of their activation. Under gabazine, the electrical stimulation of the ON or OB can induce fictive swimming—the in vitro corollary of swimming behavior. Overall, these findings suggest that the GABA_A_ antagonist gabazine increases the output of the OB. Indeed, downstream relays of the olfactomotor pathway (PT and MLR) control locomotion in a graded fashion [12,15,83]. Consequently, the increased RS cell responses observed under gabazine are likely to result from an increased drive from the OB to the PT and/or MLR. Studies in mammals have shown that GABA acts at several locations in the OB. OSN terminals express GABA_B_ receptors [84–86], which inhibit transmission from OSN axons to mitral cell primary dendrites upon release of GABA by periglomerular cells [70,71,87,88]. Mitral cell dendrites express both GABA_A_ and GABA_B_ receptors [89–93]. Pharmacological blockade or genetic alteration of GABA_A_ receptors in mitral cells alters the OB γ oscillations and leads to increased ON-induced mitral cell discharges [78,94]. The effect of GABA_B_ receptor activation in these cells is less clear [93]. Both periglomerular and granule cells release GABA on mitral cells; periglomerular cells contact mitral cell primary dendrites, whereas granule cells contact mitral cell secondary dendrites [69,95]. Granule and periglomerular cells also express GABA_A_ receptors [89,91,96,97]. Genetic alteration of the GABA_A_ receptor subtype expressed in granule cells (i.e., expressing the β3 subunit) either globally or in a cell-specific manner increases the granule cell inhibition of mitral cells and results in increased OB γ oscillations [98,99]. To the best of our knowledge, the effect of periglomerular cell GABA_A_ receptor activation on mitral cell activity has not been investigated, but an inhibition of periglomerular cells leading to the disinhibition of mitral cells could be expected. Finally, electrophysiological evidence suggests that granule cells also possess GABA_B_ receptors whose activation modulates granule cell inhibition of mitral cells [100]. GABA can thus depress or potentiate mitral cell activity depending on its site of action (i.e., OSNs axons, OB interneurons, or mitral cell). However, as OB interneurons act on mitral cells via GABA_A_ receptors, the net effect of the pharmacological blockade of GABA_A_ receptors in all OB layers is likely to be a disinhibition of mitral cells. This is consistent with our results in lampreys and those found in other vertebrate species [77,94,101–104].

The presence of both tonic and phasic inhibition in the OB has been reported in fish, amphibians, and mammals [71,77,78,94,98,99,102,105,106]. It has been suggested that tonic inhibition may modulate the strength of sensory inputs to the OB [88] or the sensitivity of second-order olfactory neurons to sensory inputs [102]. Phasic inhibition has been shown to generate neuronal synchrony (i.e., oscillations) in projection neurons [98,99]. The role of these oscillations and thus of the phasic inhibition is still debated, but several studies in insects and mammals point toward a crucial role in coding olfactory information [107–109].

Our study shows that a strong GABAergic inhibition of the OB output is present in the lamprey, one of the most basal extant vertebrate, and thus may be a common ancestral feature of the vertebrate OB.

The GABAergic modulation of the olfactomotor pathways seen in lampreys could explain some of the life stage–specific behavioral responses to olfactory cues. For instance, migratory pheromones attract only pre-spawning adult lampreys [24–26]. This is surprising because these pheromones evoke strong responses in OSNs at other life stages [110]. Somehow, the activation of OSNs only leads to locomotor responses during the pre-spawning adult life stage. Meléndez-Ferro and colleagues [43,111] have stated that the density of OB GABAergic cells declines significantly between the newly transformed and pre-spawning life stages. Whether this apparent decrease in GABAergic cell density could account for some of the life stage differences is not known at present, but it could be one plausible mechanism worth investigating.

A series of recent studies have shown that a CO_2_-mediated water acidification significantly impairs several olfactory-driven behaviors in fish, including prey tracking, predator avoidance, alarm response, and homing [112–116]. The mechanism at play has not been fully characterized yet, but it involves an alteration of the normal functioning of GABA_A_ receptors, as blocking these receptors with gabazine led to a behavioral recovery [115,117]. The authors of these studies proposed that a potentiation or a reversal of the GABA_A_ receptor function (from inhibitory to excitatory) because of changes in anionic gradients over neuronal membranes could underlie these behavioral alterations. Taken together, these studies show that GABAergic mechanisms also play a crucial role in modulating olfactomotor behaviors in fish. Further studies are needed to establish whether the neural pathways and modulatory mechanisms characterized in lampreys are also present in fish and other vertebrates.

### Two distinct olfactomotor pathways

In the present study, we showed that stimulation of the MOB under gabazine led to excitatory responses in RS cells and to locomotion. This suggests the existence of a distinct pathway from the MOB to the RS cells and the presence of a strong tonic GABAergic inhibition in the MOB. We characterized the anatomy and physiology of this pathway. Anatomical data showed that the LPal receives a massive projection from the MOB and projects down to both the PT and MLR. The PT, in turn, projects to the MLR [12,118,119]. We also showed that the MLR receives a direct projection from the medOB, in addition to the already characterized projection via the PT [12]. The MLR then reaches the command cells for locomotion, the RS cells, via glutamatergic and cholinergic projections [120–123]. Physiological data confirmed that the LPal relays MOB olfactory inputs to the RS cells via the PT and MLR. This is consistent with the recent findings of Suryaranayana and colleagues [124] indicating that some LPal neurons receive monosynaptic inputs from the OB. Interestingly, Ocaña and colleagues [48] showed that a few fibers originating in the LPal could reach RS neurons directly and that some of these could be followed as far as the first spinal segments. This prompted the authors to conclude that the LPal possesses an efferent projection pattern similar to that of the amniote motor cortex [48]. It would be interesting to examine if these projections from the LPal to the RS neurons and spinal cord are also involved in olfactomotor responses. Although we cannot exclude that there may be other pathways linking olfactory centers to motor centers, our study demonstrates the existence of two distinct glutamatergic pathways linking the olfactory and motor systems in lampreys (Fig 11). Both these pathways share a common output via the PT/MLR–RS neurons system. However, they differ regarding their pathways from the OB to motor control centers (i.e., PT/MLR), as well as to their inputs from the periphery.

In lampreys, the main olfactory epithelium contains numerous tall, ciliated OSNs expressing the G-protein G_olf_, as in the main olfactory epithelium in other vertebrates [125–133]. The OSNs of the main olfactory epithelium project their axons to the MOB, which, in turn, projects mainly to the LPal, i.e., the putative homologue of the mammalian olfactory cortex in lampreys [134]. This pathway is strikingly similar to the main olfactory pathway of terrestrial vertebrates and thus further supports its evolutionary conservation. In addition to the main olfactory epithelium, lampreys possess an accessory olfactory organ [29–32,135–138]. The accessory olfactory organ contains short, broad, ciliated OSNs [32] that do not express the G-protein G_olf_ and project only to the medOB [32,129]. Projection neurons of the medOB then project directly to the PT and MLR, bypassing the LPal. Taken together, these findings show that the lamprey accessory olfactory organ constitutes a discrete olfactory subsystem. It has even been suggested that the accessory olfactory organ represents a primordial vomeronasal system [29,31,138].

In other vertebrates, the presence of parallel olfactory pathways conveying the information from the periphery to high-order brain olfactory centers suggests that these systems subserve different behavioral functions [139–143]. For instance, in fish, segregated olfactory pathways, from the olfactory epithelium to the telencephalon, mediate feeding, reproductive, and alarm behaviors [139,142,144–151]. Similarly, the main and accessory (i.e., vomeronasal) systems of terrestrial vertebrates are segregated until at least the third-order neurons and their respective activation elicits different behaviors [152–155]. Physiological evidence in lampreys also supports this hypothesis, as OB local field recordings showed that the medOB and MOB have overlapping but different response profiles to feeding cues and pheromones [33,34]. Moreover, we show that the pathways from the OB to the motor control centers differ for the two olfactory subsystems. The medOB projects directly to motor control centers, whereas the MOB projects first to the LPal before reaching motor control centers. Not surprisingly, activation of both systems leads to locomotion. This could be attributed to the paucity of the behavioral repertoire of lampreys compared to mammals. However, it should be noted that reproductive, migratory, and feeding behaviors all require locomotion in lampreys. The distinction between these two subsystems thus lies in their inputs from the periphery (accessory olfactory organ versus main olfactory epithelium) as well as in the involvement of the LPal in the lateral pathway. It is tempting to propose that the medial pathway could mediate innate responses to chemical stimuli (for example, avoidance), whereas the lateral pathway could be involved in olfactomotor behaviors requiring further processing and perhaps learning (for example, olfactory navigation). A similar distinction between dual “olfactory” systems exists in invertebrates [156–158]. In mammals, it was shown that mitral cells of the MOB can develop differential responses to rewarded/unrewarded odors [159]. It has been suggested that the dichotomy between innate responses versus learned responses may be what distinguish the main and accessory systems of terrestrial vertebrates [154]. This hypothesis has, however, received little attention, and further studies are needed.

In conclusion, our study shows that olfactory inputs can activate the locomotor command system via two distinct glutamatergic pathways in lampreys. To the best of our knowledge, this is the first characterization of a dual olfactory pathway, from the periphery to the motor command system, in vertebrates. Both pathways are strongly modulated by the GABAergic circuitry of the OB that may account for some of the variability in behavioral responses to olfactory inputs in lampreys. The existence of two segregated olfactory subsystems in one of the most basal extant vertebrates sheds light on the evolution of the olfactory system and suggests that its organization in functional clusters could constitute a common ancestral trait of vertebrates.

## Materials and methods

### Ethics statement

For all procedures, the animals were deeply anesthetized with tricaine methanesulphonate (MS-222, 200 mg/L, Sigma-Aldrich, Oakville, ON) and then decapitated. All surgical and experimental procedures conformed to the guidelines of the Canadian Council on Animal Care and were approved by the animal care and use committee of the Université de Montréal (Protocol no. 18–018), the Université du Québec à Montréal, and the University of Windsor.

### Experimental animals and brain preparation

Experiments were performed on 57 larval and 61 adult sea lampreys (*Petromyzon marinus*) of both sexes. Some animals were used in more than one experiment. Larvae were collected from the Pike River stream (QC, Canada). Adults were collected from the Great Chazy River (NY, United States) and were kindly provided by agents of the U.S. Fish and Wildlife Service of Vermont. The permission to collect animals in the field was granted by the Quebec's Ministry of Natural Resources and Wildlife (permit no. 2017-03-30-2189-16-SP). All animals were kept in aerated fresh water maintained at 4–5 °C.

For all types of experiments, the animals were deeply anesthetized with tricaine methanesulphonate (MS-222, 200 mg/L, Sigma-Aldrich), decapitated caudal to the seventh branchiopore, and transferred into cold oxygenated Ringer's (8–10 °C) of the following composition (in mM): 130 NaCl, 2.1 KCl, 2.6 CaCl2, 1.8 MgCl2, 4.0 HEPES, 4.0 dextrose, and 1.0 NaHCO3, at pH 7.4. The branchial apparatus, myotomal musculature, and all soft tissues attached to the ventral side of the cranium were removed. The dorsal part of the vertebrae and cranium were removed to expose the brain and the rostral spinal cord. The peripheral olfactory organ was left intact with the ON still attached to the brain. All other nerves were cut and the choroid plexus covering the fourth and the mesencephalic ventricles was removed.

### Electrophysiological recordings

The preparation was pinned down to the bottom of a recording chamber lined with Sylgard (Dow Corning, Midland, MI) and continuously perfused with cold oxygenated Ringer's (about 4 mL/min). Intracellular recordings of RS neurons were performed under visual guidance through a M3C stereomicroscope (Wild-Heerbrugg, Heerbrugg, Switzerland) using sharp glass microelectrodes (60–130 MΩ) filled with 4 M potassium acetate. The signals were amplified with an Axoclamp 2A (20 kHz sampling rate, Axon Instruments, Foster City, CA) and acquired through a Digidata 1322A interface running on pClamp 9.2 software (Axon Instruments, Foster City, CA). Only RS neurons displaying a stable membrane potential lower than −70 mV for at least 15 min were considered in this study.

Electrical stimulation (1–3 pulses, 5–30 μA, 2-ms duration, and 20-ms pulse interval) was delivered using homemade glass-coated tungsten electrodes (0.8–2 MΩ, 10–50 μm tip exposure) connected to a Grass S88 stimulator via a Grass PSIU6 photoelectric isolation unit (Astro-Med, Longueuil, QC). A delay of 50 s was allowed between each stimulation. In the figures, the synaptic responses are illustrated as the mean of 10 responses obtained with the same stimulation parameters.

In some experiments, the left and right ventral roots from one spinal segment (usually around the 10th segment) were also recorded using extracellular glass electrodes (tip diameter about 5 μm) filled with the Ringer's solution. The signals were amplified (×10,000) and filtered (100 Hz–1 kHz band-pass) using an AM systems 1800 dual channel amplifier (AM systems, Sequim, WA) and monitored for the presence of neural activity. “Fictive locomotion” (originally defined by Perret and colleagues, 1972) [160] observed in the absence of muscles and movement was defined as a neural activation of the spinal ventral (motor) roots, with a similar pattern as myotomal contractions seen during swimming.

### Drug application

Drugs were purchased from Sigma-Aldrich (AP5), Tocris Bioscience (gabazine and CNQX), and Thermo Fisher Scientific (Fast Green). They were kept as frozen concentrated stock solutions and dissolved to their final concentrations in Ringer's solution prior to their use. Gabazine (SR-95531; 0.1–1 mM) and the CNQX/AP5 mixture (0.5 mM/1 mM) were pressure ejected (3–180 pulses; mean ± SD = 57 ± 46 pulses; about 4 psi, 20–40-ms pulse duration, injection volume: 0.1–6.6 nL; mean ± SD = 2.6 ± 2.1 nL) through glass micropipettes (10–20 μm tip diameter) in the brain tissue, using a Picospritzer (General Valve Corp, Fairfield, NJ). The inert dye Fast Green was added to the drug solution to monitor the extent of the injections. The spread did not exceed 300 μm in diameter for any microinjection.

### Calcium imaging experiments

RS cells were retrogradely labeled by placing crystals of the calcium-sensitive indicator dye Calcium-Green dextran (3000 MW, Invitrogen, Eugene, OR) on the rostral stump of the spinal cord, transected at the level of the first spinal segments. The preparation was then kept in cold, oxygenated Ringer's solution for 24–36 h of axonal transport. Labeled cells were observed under a Nikon epifluorescence microscope equipped with a 20× (0.75 NA) objective. A filter set appropriate for fluorescein isothiocyanate (FITC) was used to visualize the neurons. The emitted light was captured with an intensified CCD video camera (Photometrics CoolSNAP HQ, Roper Scientific, Tucson, AZ) and recorded at a rate of two images per second, using Metafluor imaging software (Molecular Devices, Sunnyvale, CA). Calcium responses are expressed as relative changes in fluorescence (ΔF/F).

### Anatomical experiments

#### Axonal tracing

Anterograde and retrograde labeling was obtained after unilateral injections of Texas Red-conjugated dextran amines (TRDA, 3000 MW, Molecular Probes, Eugene, OR) or biocytin (Sigma-Aldrich, Oakville, ON). In all cases, the site to be injected was first carefully lesioned with the fine tip of a pulled glass capillary and crystals of the tracer were immediately inserted into the lesion, all while the brain was maintained in cold, oxygenated Ringer’s. After waiting 10–15 min for the lesioned axons to pick up the dissolved tracer, the preparation was rinsed thoroughly and left in cold, oxygenated Ringer’s solution for 3–18 h to allow for the passive diffusion of the tracer in the axons. The preparation was then fixed in a solution of 4% paraformaldehyde in phosphate-buffered saline, pH 7.4, for 24 h and then transferred overnight into a solution of 20% sucrose in phosphate buffer. Transverse sections of 25-μm thickness were made with a cryostat and collected on ColorFrost Plus microscope slides (Thermo Fisher Scientific, Waltham, MA). The sections were left to dry overnight on a warming plate set at 37 °C. The following day, three changes of PBS were used to rehydrate and rinse the sections and biocytin, if present in the tissue, was revealed for 60 min with streptavidin conjugated to Alexa Fluor fluorophores of different wavelengths, depending on the needs (Streptavidin-Alexa Fluor 594, 488 or 350, dilution 1:400 in PBS, Molecular Probes, Eugene, OR). When needed, DAPI (included in the mounting medium) or Neurotrace Green (120-min incubation, diluted 1:200 in PBS at room temperature) was used as counterstain. The slides were mounted with Vectashield (H-1000 or H-1200, Vector laboratories, Burlington, ON) for observation under epifluorescence microscopy (E600 microscope equipped with a DXM1200 digital camera, Nikon Canada, Mississauga, ON).

#### Immunofluorescence and lectin binding

When immunofluorescence against GABA and/or lectin binding (to label primary olfactory afferents) was performed after axonal tracing, the brain preparation was immersed for 4 h in the fixative solution aforementioned, but with the addition of 2% glutaraldehyde. After thorough rinsing in PBS, the brain was sectioned on a Vibratome at 40-μm thickness and the sections were collected in PBS and processed for biocytin revelation, lectin binding, and immunofluorescence, as follows. First, the sections were incubated for 1 h in the incubation medium (PBS containing 5% normal goat serum, 0.3% Triton X-100) containing streptavidin-Alexa Fluor 350 (diluted 1:200, Invitrogen, Eugene, OR). The sections were rinsed three times for 10 min with the incubation medium, and then the primary antibody was added for an incubation of 24–36 h at 4 °C (monoclonal mouse anti-GABA, diluted 1:5,000, see below). The following day, the sections were rinsed three times for 10 min with PBS and incubated for 1 h in the incubation medium containing a goat anti-mouse-DyLight 594 secondary antibody (dilution: 1:400, Jackson ImmunoResearch Laboratories, West Grove, PA). The sections were then rinsed three times for 10 min with PBS and incubated for 1 h in PBS containing the isolectine GS-IB4-Alexa Fluor 488 (diluted 1:100, Invitrogen, Eugene, OR). The sections were rinsed once again three times for 10 min with PBS, then rinsed quickly with distilled water and mounted with Vectashield (H-1000, Vector Laboratories, Burlington, ON).

The monoclonal anti-GABA antibody, clone 3A12, was kindly donated by Dr. Peter Streit, Zürich, Switzerland, and was successfully used on the lamprey brain tissue in previous studies [118,161]. It was developed following immunization with GABA conjugated to BSA with glutaraldehyde (GABA-G-BSA) and has been well characterized [162]. Briefly, this clone was shown by enzyme-linked immunosorbent assay (ELISA) to bind strongly to GABA-G-BSA. Immunoreactivity of the same clone to β-alanine-G-BSA was 4,000 times less, and even lower for glycine-, aspartate-, glutamine-, and taurine-G-BSA. Immunoreactivity of the clone to BSA or G-BSA was almost nonexistent, and preabsorption with GABA-G-BSA completely abolished the immunohistochemical staining of rat brain sections. Double-labeling experiments using the mAb 3A12 and a commercially available rabbit anti-GABA antibody (1:100; Cat. No. AB131; Chemicon, Temecula, CA) showed immunoreactivity in the same cell populations in the lamprey brain. The AB131 antibody was developed by immunization of rabbits with GABA-G-BSA and tested for specificity on rat and human cerebellum. The immunostaining was completely abolished by preincubation of the antibody with 10–100 μg of GABA-G-BSA per mL of diluted antibody. Furthermore, spinal cord sections incubated with mAb 3A12 revealed the same pattern of immunostaining as previously shown [163,164]. No immunoreactivity was detected when the primary antibody was omitted from the immunohistochemical processing.

### Data and statistics

Results are presented as mean ± SD. Statistical analyses were performed using Sigma Plot 11.0 (Systat, San Jose, CA), and statistical significance was set at *p* < 0.05. To test for differences in mean between groups, we performed a one-way analysis of variance for repeated measures, followed by a Holm-Sidak’s multiple-comparison post hoc test or Friedman analysis of variance for repeated measures on ranks followed by Tukey multiple-comparison post hoc test.

## Supporting information

S1 Fig Relative depth of OB GABAergic and projection neurons. Bar graph showing the distribution of GABAergic (red) and projection (blue) neurons in the olfactory bulb. There is an overlap between the two populations, but the mean relative depth of GABAergic and projection neurons is significantly different (*p* < 0.001). Note that there are no GABAergic neurons in the most peripheral region of the OB and nearly no projection neurons in the most central region of the OB. The numerical values underlying this figure can be found in S1 Data. OB, olfactory bulb.
(TIF)

S2 Fig medOB neurons projecting to the PT are approached by primary afferent and GABAergic processes. (A) Schematic illustration of the brain showing the tracer injection site and the level of the cross sections shown in B and C. (B) Projection neurons (blue) in medOB are labeled from an injection of biocytin in the PT. The olfactory primary afferent fibers are labeled with GSIB4 (green), and processes and cell bodies containing GABA were labeled by immunofluorescence (red). (C) High-power confocal image from an area corresponding to the white frame in B. The image was taken from an adjacent section in the same animal and is the result of a z-projection of two 1-μm optical sections from a z-stack. Scale bar in B = 100 μm; scale bar in C = 10 μm. Di, diencephalon; GSIB4, *Griffonia simplicifolia* isolectin B4; LPal, lateral pallium; medOB, medial part of the olfactory bulb; Mes, mesencephalon; MOB, main olfactory bulb; OB, olfactory bulb; PT, posterior tuberculum.
(TIF)

S3 Fig RS neurons from all four reticular nuclei respond similarly to ON stimulation. (A) Schematic illustration of the brain showing stimulation and imaging sites. (B1–4) Photomicrographs of calcium green–loaded RS neurons in the four reticular nuclei; the mesencephalic (MRN, B1), anterior rhombencephalic (ARRN, B2), middle rhombencephalic (MRRN, B3), and posterior rhombencephalic (PRRN, B4) reticular nuclei. (C1–4) Calcium responses (ΔF/F) of RS neurons from the MRN, ARRN, MRRN, and PRRN to repetitive ON stimulation (5–15 μA, 5–10 Hz, 10 s, arrows) before (C1–C4) and after a local injection of gabazine (1 mM) in the OB (D1–D4). The mean calcium response (ΔF/F) of RS neurons is significantly increased in all four reticular nuclei following the injection of gabazine (*p* < 0.001,). The numerical values underlying this figure can be found in S1 Data. Note that the pseudocolors here correspond to the change in fluorescence intensity (ΔF/F) according to the scale to the right, whereas the pseudocolors in B1–4 correspond to the intensity of the initial labeling in neurons. ARRN, anterior rhombencephalic reticular nucleus; Di, diencephalon; LPal, lateral pallium; medOB, medial part of the olfactory bulb; Mes, mesencephalon; MOB, main olfactory bulb; MRN, mesencephalic reticular nucleus; MRRN, middle rhombencephalic reticular nucleus; OB, olfactory bulb; ON, olfactory nerve; PRRN, posterior rhombencephalic reticular nucleus; PT, posterior tuberculum; Rh, rhombencephalon; RS, reticulospinal.
(TIF)

S4 Fig Bath application of gabazine increases OB neuron responses to ON stimulation. (A) Schematic illustration of the rostral part of the brain showing stimulation and recording sites. The meninges and the dorsalmost layer of the OB were removed with a Vibratome to gain access directly to the glomerular layer. (B) Representative examples of extracellular recordings of the OB. A bath application of gabazine (10 μM) increases the OB neuron responses (arrows) to ON electrical stimulation (5 μA). Top traces: rectified-integrated signals; bottom traces: raw signals; asterisks indicate stimulation artifacts. (C) Average OB neuron responses (normalized amplitude of the rectified-integrated signals) to ON stimulation over time in six preparations. (D) Univariate scatterplot showing the normalized (as a percentage of control) OB neuron response for all animals. The average OB neuron response is significantly increased after a bath application of 10 μM gabazine (*n* = 6; 10 stimulations per condition per preparation). An asterisk (*) indicates a statistically significant difference at the level *p* < 0.05, while n.s. indicates the absence of statistically significant difference. The numerical values underlying this figure can be found in S1 Data. Di, diencephalon; LPal, lateral pallium; Mes, mesencephalon; OB, olfactory bulb; ON, olfactory nerve.
(TIF)

S5 Fig Electrical stimulation of the MOB elicits responses in RS neurons after resection of the medOB. (A) Schematic illustration of the brain showing stimulation, injection, and recording sites. The dashed area represents the medOB resection. (B) Responses of a RS neuron to the electrical stimulation of the MOB (15 μA) after a colocalized injection of gabazine (0.1 mM). The black trace is a mean of 10 individual responses (colored traces). Di, diencephalon; LPal, lateral pallium; Mes, mesencephalon; medOB, medial part of the olfactory bulb; MOB, main olfactory bulb; Rh, rhombencephalon; RS, reticulospinal.
(TIF)

S6 Fig The LPal in lampreys. (A-D) Photomicrographs of cross sections illustrating the general cytoarchitecture of the LPal, from rostral (A) to caudal (D). The DAPI (a blue fluorescent DNA dye) photomicrographs were inverted and converted to gray scale. The LPal was defined here as the lateral part of the evaginated telencephalon where many neurons are typically organized in many small clusters. The consistency in morphology and size of the retrogradely labeled neurons, especially those projecting to the PT, also helped in defining the extent of the LPal. This was particularly useful in the rostral part (A), where retrogradely labeled cells from the PT were only found in the ventrolateral area. Scale bar in A for all photomicrographs = 200 mm. dmtn, dorsomedial telencephalic nucleus; LPal, lateral pallium; OB, olfactory bulb; PT, posterior tuberculum.
(TIF)

S7 Fig The lateral olfactomotor pathway is also present in larvae. (A) Schematic illustration showing the tracer injection in the PT and the level of the cross section photographed in B. The injection site in the PT is also illustrated in a photomicrograph of a cross section. (B1) Many neurons were retrogradely labeled (red) in the LPal from an injection of biocytin in the PT. The meningeal cells around the LPal show strong red autofluorescence (asterisks). (B2) The retrogradely labeled neurons from B1 superimposed over a Nissl stain (green). (C) Schematic illustration showing tracer injections and the level of the cross section photographed in D. The injection site in the MLR is also illustrated in a photomicrograph of a cross section; note that the dorsal midline at the isthmus was sectioned to gain access to the MLR. (D1) A few neurons were retrogradely labeled (arrows) in the LPal after an injection of biocytin in the MLR. The meningeal cells around the LPal show strong green autofluorescence (asterisks). (D2) The retrogradely labeled neurons from D1 superimposed over a DNA-labeling DAPI stain. All scale bars = 100 μm. LPal, lateral pallium; MLR, mesencephalic locomotor region; OB, olfactory bulb; PT, posterior tuberculum.
(TIF)

S1 Data Numerical values underlying charts and statistical tests. (XLSX)

## Abbreviations

AP5: 2-amino-5-phosphonopentanoic acid
CNQX: 6-cyano-7-nitroquinoxaline-2,3-dione
CNS: central nervous system
cVR: contralateral ventral root
fr: fasciculus retroflexus
GABA-G-BSA: GABA conjugated to BSA with glutaraldehyde
GL: glomerular layer
GSIB4: *Griffonia simplicifolia* isolectin B4
ICL: internal cell layer
iVR: ipsilateral ventral root
LPal: lateral pallium
medOB: medial part of the olfactory bulb
MLR: mesencephalic locomotor region
MOB: main olfactory bulb
NMLF: nucleus of the medial longitudinal fasciculus
OB: olfactory bulb
ON: olfactory nerve
ONL: olfactory nerve layer
OSN: olfactory sensory neuron
PT: posterior tuberculum
Rh: rhombencephalon
RS: reticulospinal
TRDA: Texas Red-conjugated dextran amine
