# Supplementary figures and images for: GABAergic modulation of olfactomotor transmission in lampreys
Source: PLoS Biol. 2018 Oct 4;16(10):e2005512. doi: 10.1371/journal.pbio.2005512 (PMC6191151; doi:10.1371/journal.pbio.2005512)

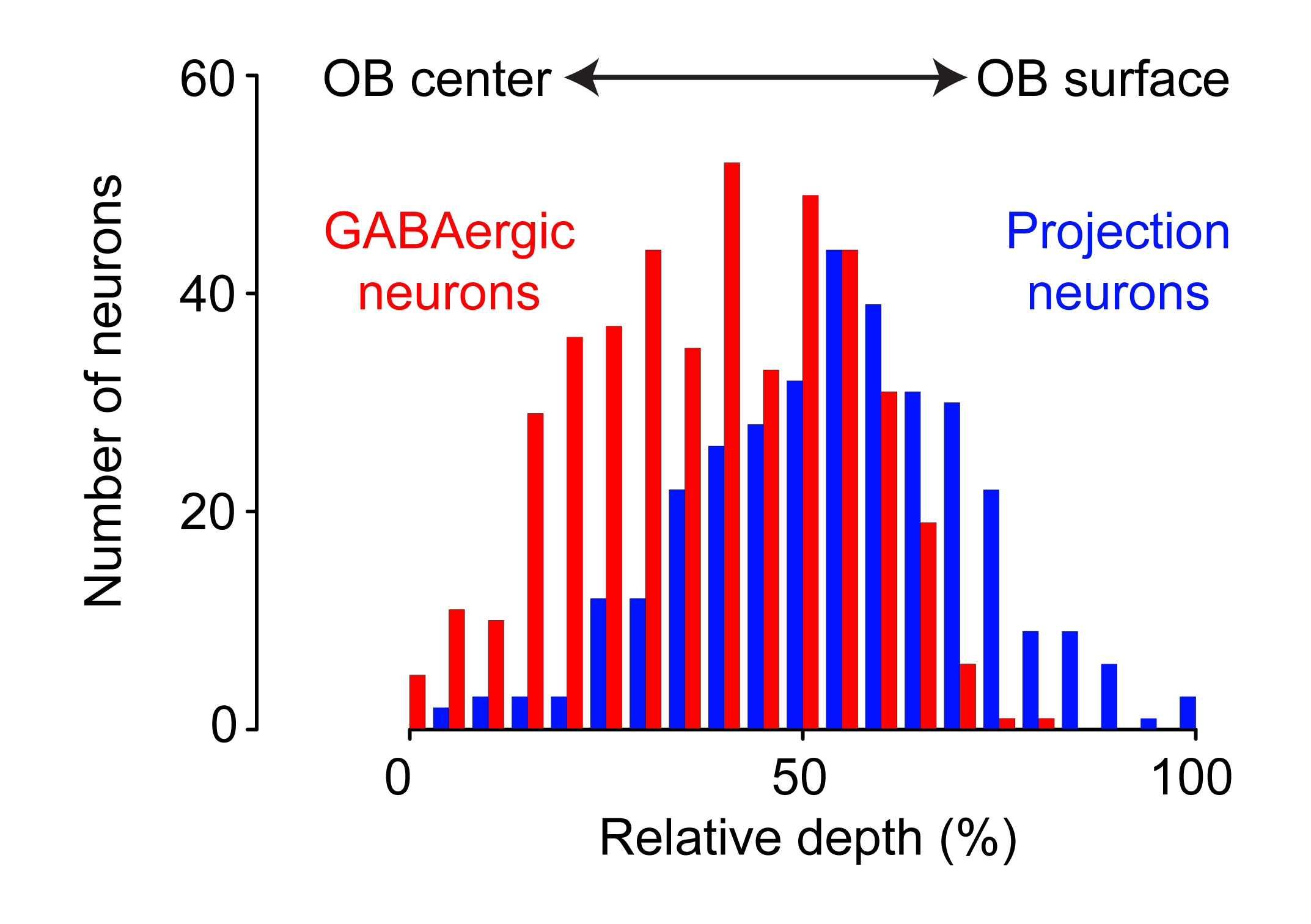

Supplement: S1 Fig — Bar graph showing the distribution of GABAergic (red) and projection (blue) neurons in the olfactory bulb. There is an overlap between the two populations, but the mean relative depth of GABAergic and projection neurons is significantly different (p < 0.001). Note that there are no GABAergic neurons in the most peripheral region of the OB and nearly no projection neurons in the most central region of the OB. The numerical values underlying this figure can be found in S1 Data. OB, olfactory bulb. (TIF) [file pbio.2005512.s001.tif]

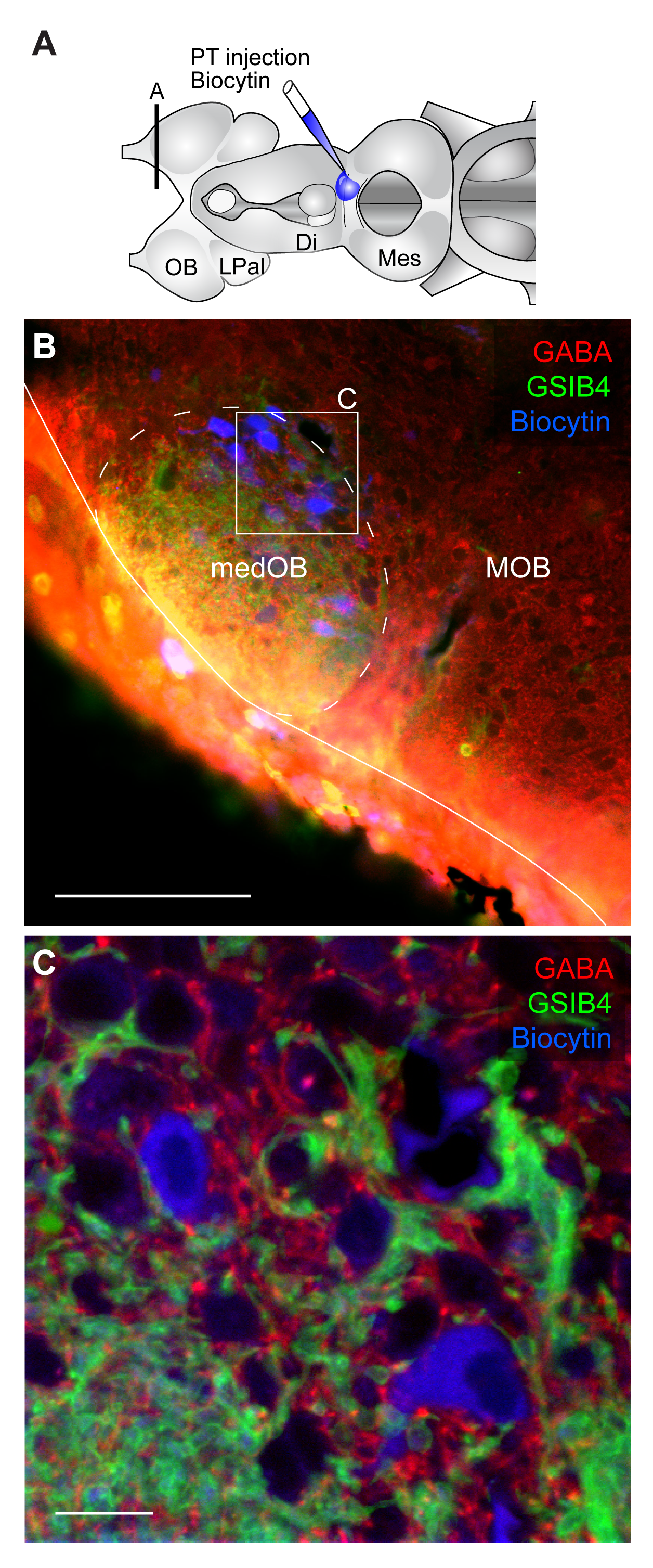

Supplement: S2 Fig — (A) Schematic illustration of the brain showing the tracer injection site and the level of the cross sections shown in B and C. (B) Projection neurons (blue) in medOB are labeled from an injection of biocytin in the PT. The olfactory primary afferent fibers are labeled with GSIB4 (green), and processes and cell bodies containing GABA were labeled by immunofluorescence (red). (C) High-power confocal image from an area corresponding to the white frame in B. The image was taken from an adjacent section in the same animal and is the result of a z-projection of two 1-μm optical sections from a z-stack. Scale bar in B = 100 μm; scale bar in C = 10 μm. Di, diencephalon; GSIB4, Griffonia simplicifolia isolectin B4; LPal, lateral pallium; medOB, medial part of the olfactory bulb; Mes, mesencephalon; MOB, main olfactory bulb; OB, olfactory bulb; PT, posterior tuberculum. (TIF) [file pbio.2005512.s002.tif]

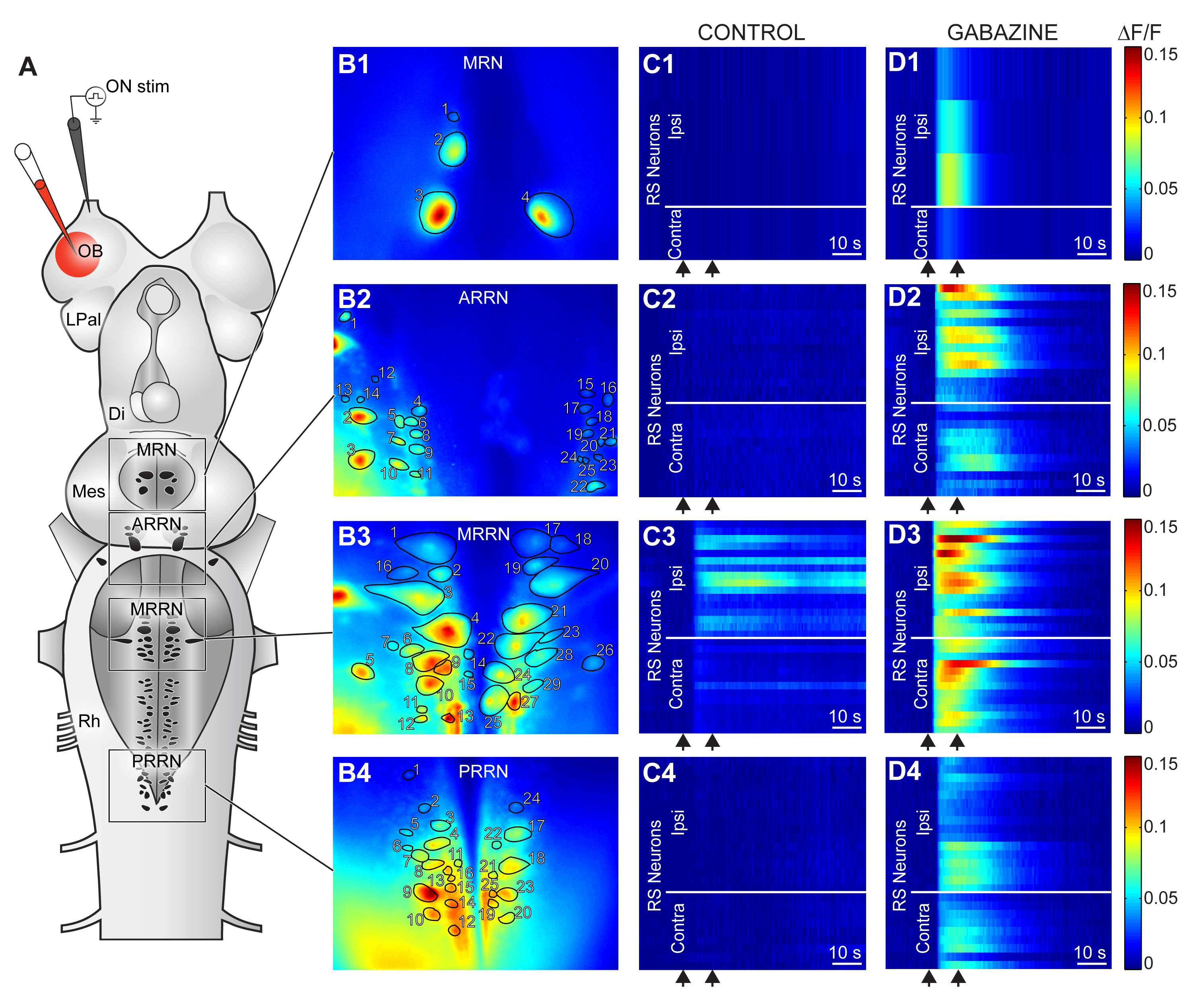

Supplement: S3 Fig — (A) Schematic illustration of the brain showing stimulation and imaging sites. (B1–4) Photomicrographs of calcium green–loaded RS neurons in the four reticular nuclei; the mesencephalic (MRN, B1), anterior rhombencephalic (ARRN, B2), middle rhombencephalic (MRRN, B3), and posterior rhombencephalic (PRRN, B4) reticular nuclei. (C1–4) Calcium responses (ΔF/F) of RS neurons from the MRN, ARRN, MRRN, and PRRN to repetitive ON stimulation (5–15 μA, 5–10 Hz, 10 s, arrows) before (C1–C4) and after a local injection of gabazine (1 mM) in the OB (D1–D4). The mean calcium response (ΔF/F) of RS neurons is significantly increased in all four reticular nuclei following the injection of gabazine (p < 0.001,). The numerical values underlying this figure can be found in S1 Data. Note that the pseudocolors here correspond to the change in fluorescence intensity (ΔF/F) according to the scale to the right, whereas the pseudocolors in B1–4 correspond to the intensity of the initial labeling in neurons. ARRN, anterior rhombencephalic reticular nucleus; Di, diencephalon; LPal, lateral pallium; medOB, medial part of the olfactory bulb; Mes, mesencephalon; MOB, main olfactory bulb; MRN, mesencephalic reticular nucleus; MRRN, middle rhombencephalic reticular nucleus; OB, olfactory bulb; ON, olfactory nerve; PRRN, posterior rhombencephalic reticular nucleus; PT, posterior tuberculum; Rh, rhombencephalon; RS, reticulospinal. (TIF) [file pbio.2005512.s003.tif]

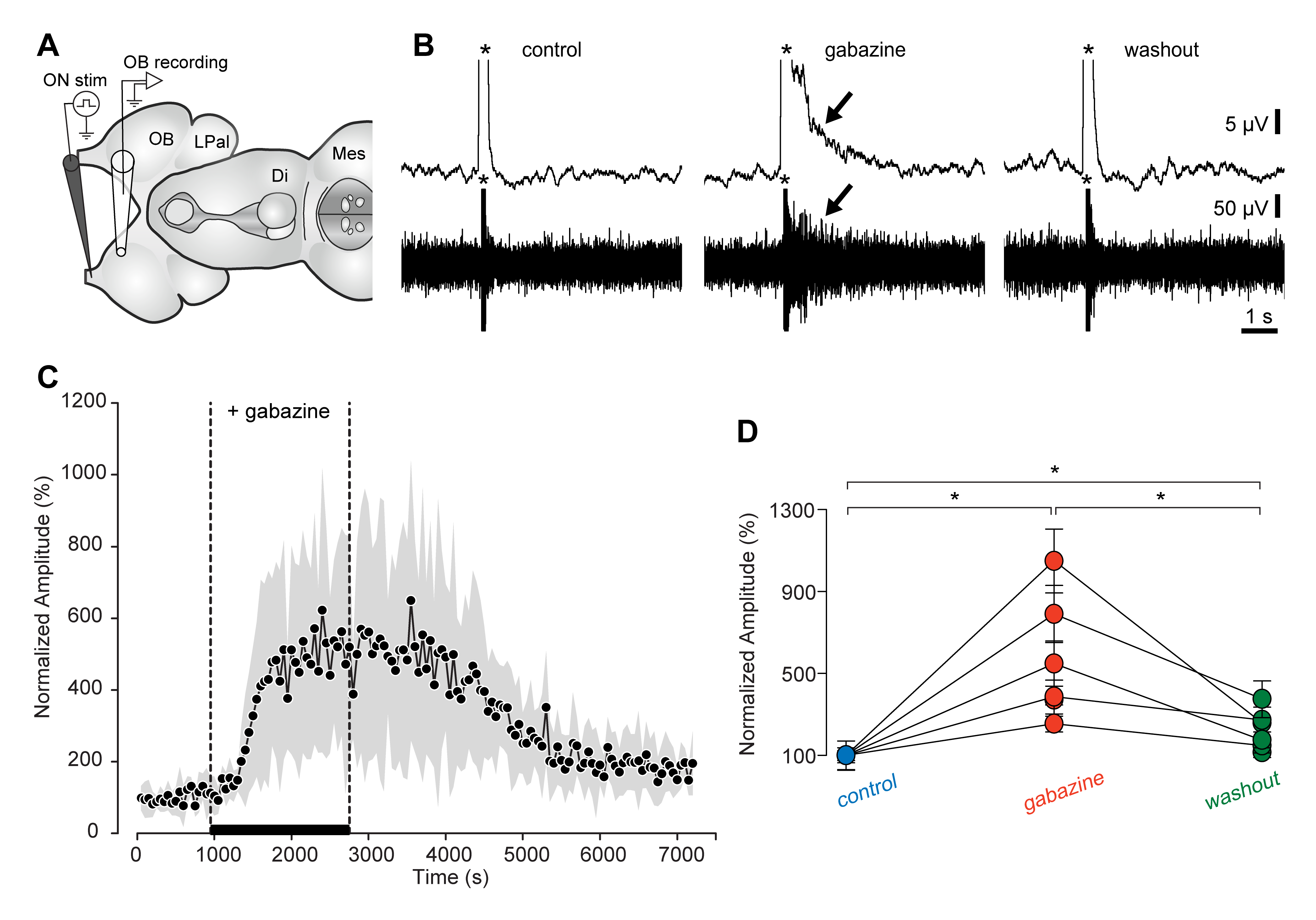

Supplement: S4 Fig — (A) Schematic illustration of the rostral part of the brain showing stimulation and recording sites. The meninges and the dorsalmost layer of the OB were removed with a Vibratome to gain access directly to the glomerular layer. (B) Representative examples of extracellular recordings of the OB. A bath application of gabazine (10 μM) increases the OB neuron responses (arrows) to ON electrical stimulation (5 μA). Top traces: rectified-integrated signals; bottom traces: raw signals; asterisks indicate stimulation artifacts. (C) Average OB neuron responses (normalized amplitude of the rectified-integrated signals) to ON stimulation over time in six preparations. (D) Univariate scatterplot showing the normalized (as a percentage of control) OB neuron response for all animals. The average OB neuron response is significantly increased after a bath application of 10 μM gabazine (n = 6; 10 stimulations per condition per preparation). An asterisk (*) indicates a statistically significant difference at the level p < 0.05, while n.s. indicates the absence of statistically significant difference. The numerical values underlying this figure can be found in S1 Data. Di, diencephalon; LPal, lateral pallium; Mes, mesencephalon; OB, olfactory bulb; ON, olfactory nerve. (TIF) [file pbio.2005512.s004.tif]

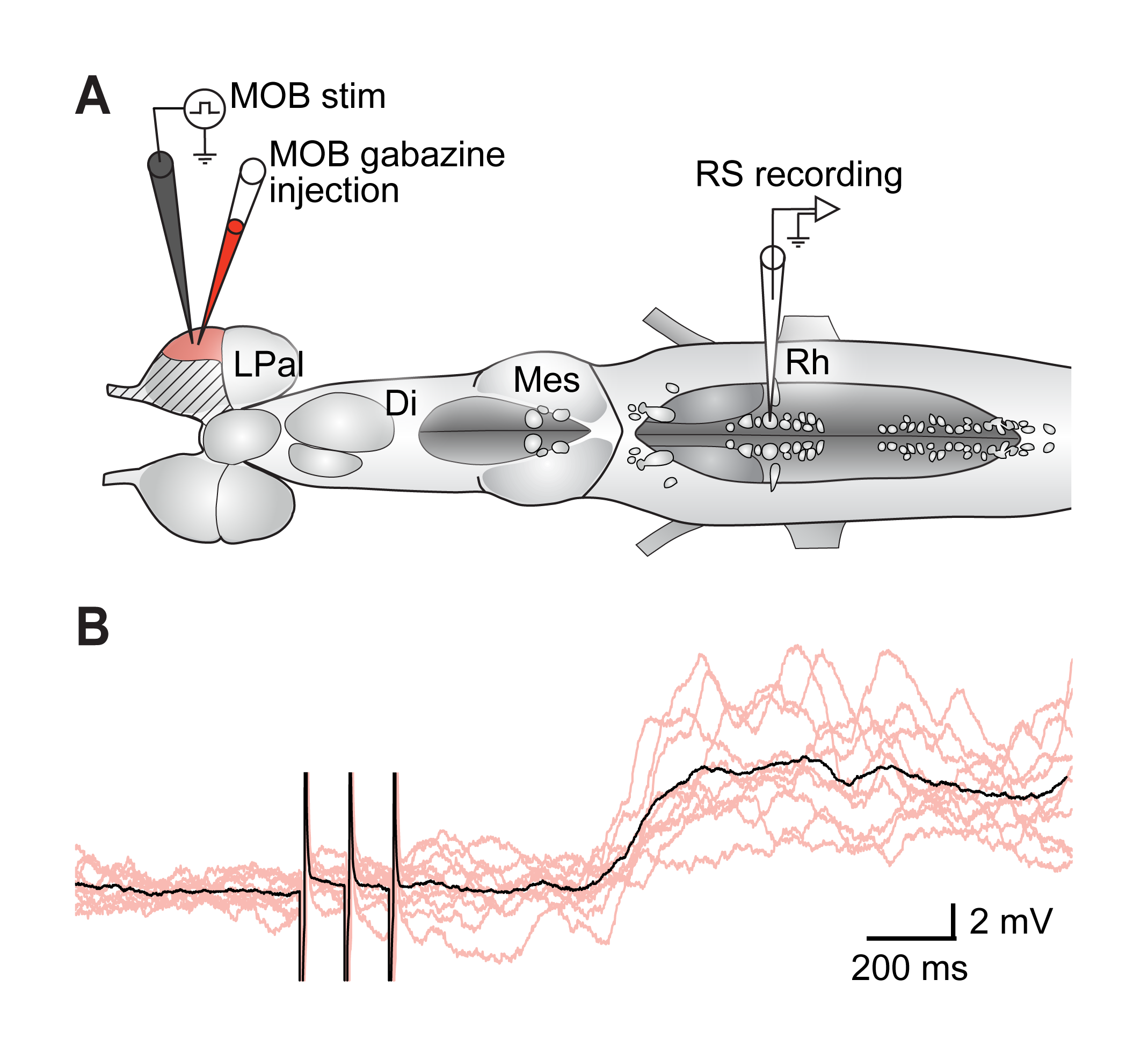

Supplement: S5 Fig — (A) Schematic illustration of the brain showing stimulation, injection, and recording sites. The dashed area represents the medOB resection. (B) Responses of a RS neuron to the electrical stimulation of the MOB (15 μA) after a colocalized injection of gabazine (0.1 mM). The black trace is a mean of 10 individual responses (colored traces). Di, diencephalon; LPal, lateral pallium; Mes, mesencephalon; medOB, medial part of the olfactory bulb; MOB, main olfactory bulb; Rh, rhombencephalon; RS, reticulospinal. (TIF) [file pbio.2005512.s005.tif]

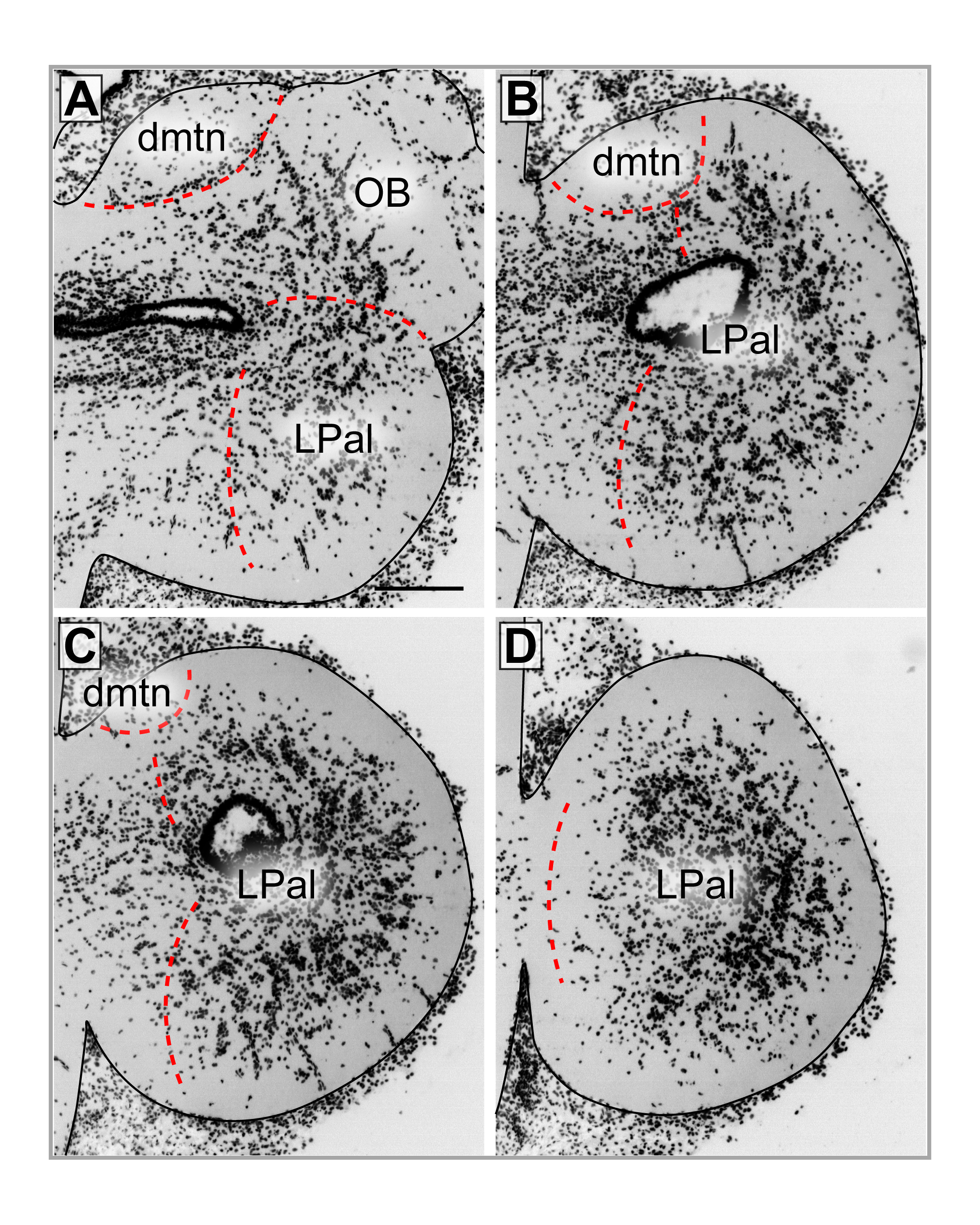

Supplement: S6 Fig — (A-D) Photomicrographs of cross sections illustrating the general cytoarchitecture of the LPal, from rostral (A) to caudal (D). The DAPI (a blue fluorescent DNA dye) photomicrographs were inverted and converted to gray scale. The LPal was defined here as the lateral part of the evaginated telencephalon where many neurons are typically organized in many small clusters. The consistency in morphology and size of the retrogradely labeled neurons, especially those projecting to the PT, also helped in defining the extent of the LPal. This was particularly useful in the rostral part (A), where retrogradely labeled cells from the PT were only found in the ventrolateral area. Scale bar in A for all photomicrographs = 200 mm. dmtn, dorsomedial telencephalic nucleus; LPal, lateral pallium; OB, olfactory bulb; PT, posterior tuberculum. (TIF) [file pbio.2005512.s006.tif]

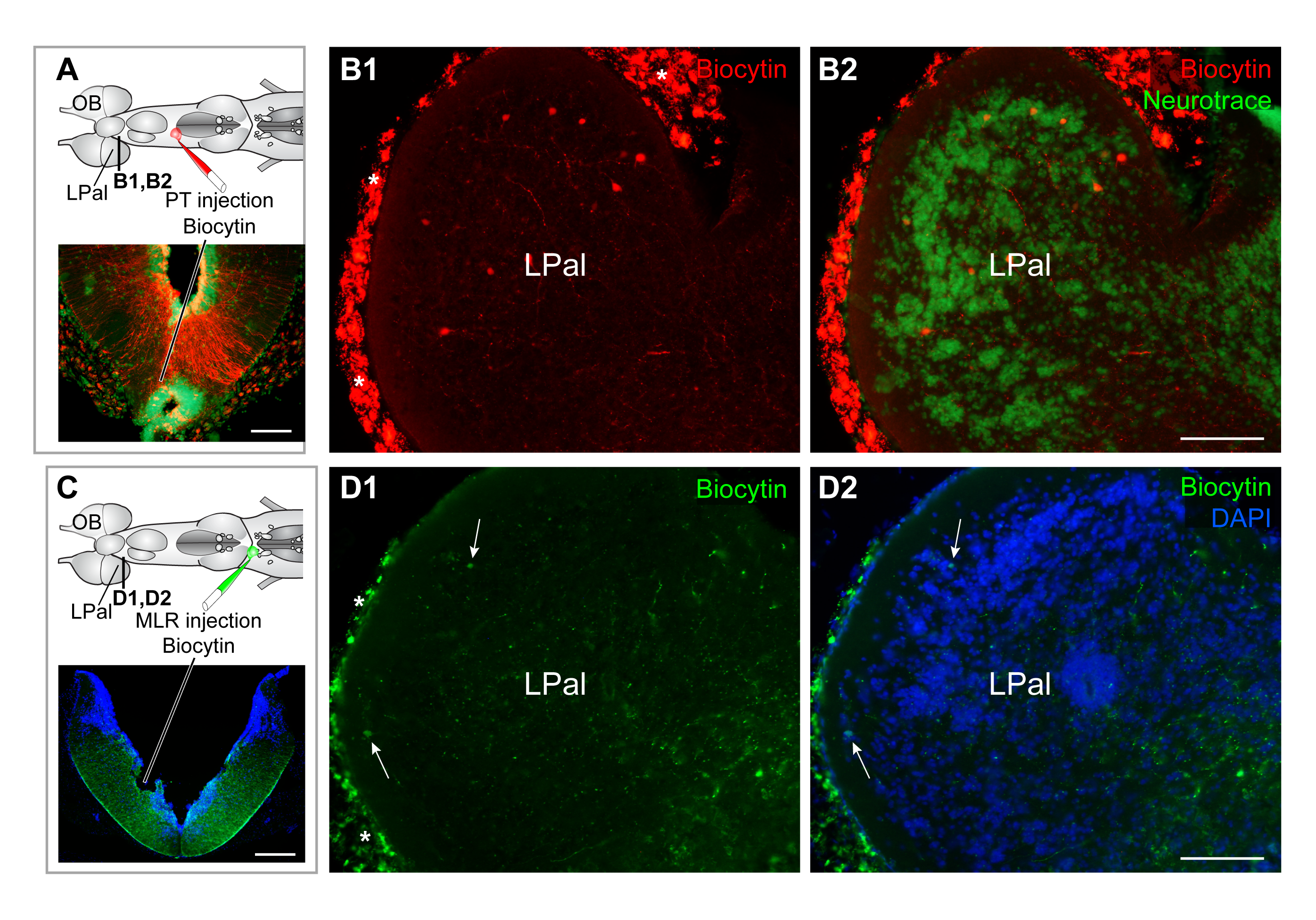

Supplement: S7 Fig — (A) Schematic illustration showing the tracer injection in the PT and the level of the cross section photographed in B. The injection site in the PT is also illustrated in a photomicrograph of a cross section. (B1) Many neurons were retrogradely labeled (red) in the LPal from an injection of biocytin in the PT. The meningeal cells around the LPal show strong red autofluorescence (asterisks). (B2) The retrogradely labeled neurons from B1 superimposed over a Nissl stain (green). (C) Schematic illustration showing tracer injections and the level of the cross section photographed in D. The injection site in the MLR is also illustrated in a photomicrograph of a cross section; note that the dorsal midline at the isthmus was sectioned to gain access to the MLR. (D1) A few neurons were retrogradely labeled (arrows) in the LPal after an injection of biocytin in the MLR. The meningeal cells around the LPal show strong green autofluorescence (asterisks). (D2) The retrogradely labeled neurons from D1 superimposed over a DNA-labeling DAPI stain. All scale bars = 100 μm. LPal, lateral pallium; MLR, mesencephalic locomotor region; OB, olfactory bulb; PT, posterior tuberculum. (TIF) [file pbio.2005512.s007.tif]
